# Role of feeding strategy bundle with acid-suppressive therapy in infants with esophageal acid reflux exposure: a randomized controlled trial

**DOI:** 10.1038/s41390-020-0932-4

**Published:** 2020-05-07

**Authors:** Sudarshan R. Jadcherla, Kathryn A. Hasenstab, Lai Wei, Erika K. Osborn, Sreekanth Viswanathan, Ish K. Gulati, Jonathan L. Slaughter, Carlo Di Lorenzo

**Affiliations:** 1 Innovative Infant Feeding Disorders Research Program, Nationwide Children’s Hospital, Columbus, OH USA; 2 Center for Perinatal Research, Abigail Wexner Research Institute at Nationwide Children’s Hospital, Columbus, OH USA; 3 Division of Neonatology, Nationwide Children’s Hospital, Columbus, OH USA; 4 Department of Pediatrics, College of Medicine, The Ohio State University College of Medicine, Columbus, OH USA; 5 Division Pediatric Gastroenterology, Hepatology and Nutrition; Department of Pediatrics, Nationwide Children’s Hospital, Columbus, OH USA; 6 Center for Biostatistics, Department of Biomedical Informatics, The Ohio State University College of Medicine, Columbus, OH USA; 7 Division of Epidemiology, College of Public Health, The Ohio State University, Columbus, OH USA

## Abstract

**Objective:**

To test the hypothesis that a feeding bundle concurrent with acid suppression is superior to acid suppression alone in improving gastroesophageal reflux disease (GERD) attributed-symptom scores and feeding outcomes in neonatal ICU infants.

**Methods:**

Infants (*N* = 76) between 34 and 60 weeks’ postmenstrual age with acid reflux index > 3% were randomly allocated to study (acid-suppressive therapy + feeding bundle) or conventional (acid-suppressive therapy only) arms for 4 weeks. Feeding bundle included: total fluid volume < 140 mL/kg/day, fed over 30 min in right lateral position, and supine postprandial position. Primary outcome was independent oral feeding and/or ≥6-point decrease in symptom score (I-GERQ-R). Secondary outcomes included growth (weight, length, head circumference), length of hospital stay (LOHS, days), airway (oxygen at discharge), and developmental (Bayley scores) milestones.

**Results:**

Of 688 screened: 76 infants were randomized and used for the primary outcome as intent-to-treat, and secondary outcomes analyzed for 72 infants (*N* = 35 conventional, *N* = 37 study). For study vs. conventional groups, respectively: (a) 33% (95% CI, 19−49%) vs. 44% (95% CI, 28−62%), *P* = 0.28 achieved primary outcome success, and (b) secondary outcomes did not significantly differ (*P* > 0.05).

**Conclusions:**

Feeding strategy modifications concurrent with acid suppression are not superior to PPI alone in improving GERD symptoms or discharge feeding, short-term and long-term outcomes.

**Impact:**

- Conservative feeding therapies are thought to modify GERD symptoms and its consequences. However, in this randomized controlled trial in convalescing neonatal ICU infants with GERD symptoms, when controlling for preterm or full-term birth and severity of esophageal acid reflux index, the effectiveness of acid suppression plus a feeding modification bundle (volume restriction, intra- and postprandial body positions, and prolonged feeding periods) vs. acid suppression alone, administered over a 4-week period was not superior in improving symptom scores or feeding outcomes.
- Restrictive feeding strategies are of no impact in modifying GERD symptoms or clinically meaningful outcomes. Further studies are needed to define true GERD and to identify effective therapies in modifying pathophysiology and outcomes.
- The improvement in symptoms and feeding outcomes over time irrespective of feeding modifications may suggest a maturational effect. This study justifies the use of placebo-controlled randomized clinical trial among NICU infants with objectively defined GERD.

## Introduction

Differentiating gastroesophageal reflux (physiological, GER) from GER disease (pathological, GERD) remains a challenge in ICU infants.^1–5^ Troublesome symptoms^6^ often trigger a battery of empiric therapies, such as acid suppression, feeding modifications, and positional changes.^7–10^ Prevalence of GERD ranges from 2 to 30% across neonatal intensive care unit (NICU)s in the United States, along with a 13-fold variation in therapies, imposing an additional economic burden of over $70K per admission and 30 hospital days.^9–12^

The infant GER questionnaire-revised (I-GERQ-R) is a survey of parental/provider perception of symptom burden thought to be due to GERD, with a 6-point decrease indicating clinical improvement.^13^ Although prior clinical trials for GERD pharmacotherapy have used symptom-based criteria,^14–17^ few have evaluated the effectiveness of a bundled holistic approach, i.e., a combination of pharmacologic-, feeding-, and positional approaches in NICU patients. Improvement of parental perception of symptoms and total GER events with left lateral position and proton pump inhibitor (PPI),^8^ reduction of GER events with infants in prone or left lateral post-prandially,^18^ and conservative strategies for 2 weeks showed improvement with I-GERQ-R scores among 1−10 months age.^19^ We observed that decreased feeding volume and prolonged feeding duration were associated with reduced GER events.^20^ However, a bundled approach combining targeted acid suppression (limited duration), feeding modifications (volume, position, duration) and postprandial positions has not been rigorously examined in infants with proven esophageal acid reflux index (ARI) severity.

Based on this rationale, we have undertaken this clinical trial to determine the effective therapeutic strategies on the clinically meaningful primary outcomes in infants presenting with GERD symptoms who have qualifying ARI criteria. The objective of this RCT was to examine the short- and long-term clinical outcomes among infants treated concurrently over 4 weeks with PPI with randomly assigned feeding strategy modifications while controlling for gestational maturity (preterm or full-term at birth) and severity of esophageal ARI (3–7%, >7%). Our hypothesis was that the study approach (acid suppression, modified feeding volume, duration, and position) was superior in achieving independent oral feeding or a 6-point reduction in I-GERQ-R vs. the conventional (acid suppression alone) feeding approach.

## Patients and methods

### Study design and experimental protocol

This is a single-center, single-blinded RCT (Clinicaltrials.gov: NCT02486263) comparing the effectiveness of two feeding strategies combined with the use of a PPI (omeprazole) to manage acid-GERD (GERD Management and Therapy trial (GMT trial)). This study was approved by the Institutional Research Board (IRB) at Nationwide Children’s Hospital, Columbus, OH (IRB # 11-00734). Omeprazole is commonly used off-label in this population within the standard of care.^9^ Data safety monitoring plan was implemented and monitored quarterly by the Data Safety Monitoring Board (DSMB). Written, signed, and informed parental consent was obtained. Health Insurance Portability & Accountability was followed. Study PI and RN coordinators were available 24/7.

Twenty-four-hour pH-impedance studies were performed^6,21,22^ (Laborie Medical Technologies, Mississauga, ON, Canada). ARI (duration of esophageal acid exposure, %) was calculated.^23^ I-GERQ-R symptom score^13,16,24^ was collected. Demographic and clinical outcomes were managed using research electronic data capture tools (REDCap) tools^25^ for up to 2 years from subject enrollment.

### Participant selection, randomization and allocation

Inclusion criteria were: (a) infants admitted with clinical symptoms of GERD between 34 and 60 weeks’ postmenstrual age, with physician’s intent to treat with acid-suppressive therapy, (b) an intake volume of full enteral feeds ≥150 mL/kg/day, (c) room air or supplemental oxygen ≤1 L per minute, and (d) ARI ≥ 3%.^6,21–23^ Exclusion criteria were: (a) infants with known genetic, metabolic or syndromic diseases; (b) neurological diseases including ≥grade III intraventricular hemorrhage or perinatal asphyxia, (c) GI malformations or surgical GI conditions, and (d) infants on acid-suppressive medication at initial evaluation. Randomization was performed among consented subjects who were stratified 1:1 ratio by ARI severity (3−7%: indeterminate acid reflux and >7%: severe acid reflux) and by birth gestation (preterm, full-term) into study feeding approach or conventional approach. Permuted block randomization with block sizes of 2, 4, 6, and 8 was undertaken by the study statistician (OSU Center for Biostatistics) using a computer-generated allocation and implemented in REDCap. Nurse coordinator enrolled subjects by verifying eligibility, obtaining parental consent, and entering demographic data into REDCap. PI and study staff who evaluated subject clinical outcomes were blinded to study allocation.

### Study interventions

#### Interventions

Providers employ uniform feeding and nutritional practices in our NICU infants as per our standardized guidelines, which applies to nutrient and volume modifications. However, upon randomization and allocation, individual protocols are complied with. Upon completion of screening, enrollment and randomization, the assigned feeding management strategy was relayed to parents and the medical team. Subjects in both arms received omeprazole off-label, as a therapeutic choice^26,27^ at a recommended dose of 0.75 mg/kg/dose BID.^14,26,28^ The conventional approach was to not adjust provider-recommended feeding strategies (i.e. fed in any position, duration, volume, and postprandial position). The study approach utilized a modified feeding strategy including: (a) feeding in the right lateral position to facilitate intraprandial gastric emptying,^29^ (b) feeding duration of at least 30 min utilizing pacing when orally fed to ensure completion of prescribed volumes or via pump to ensure steady delivery of milk if gavage-fed,^20^ (c) supine postprandial position,^30^ and (d) limiting total feeding volume to ≤140 mL/kg/day.^20^

### Outcome measures

The a priori primary end-point was achieving independent oral feeds and/or a 6-point decrease in I-GERQ-R score at 5 weeks or sooner, whichever was earliest at discharge. To clarify further, there were two scenarios: (1) Among infants who were transitioning to oral feeds (gavage-fed) at inception: success was defined as achieving full oral feeds or a >6-point decrease from baseline I-GERQ-R. (2) Among infants who were on full oral feeds at inception, success was determined if full oral feeds were maintained plus a ≥6-point decrease from baseline I-GERQ-R. Secondary end-points included growth metrics (weight, length and head circumference), supplemental oxygen, economic metrics (LOHS), long-term feeding outcomes at 6 months and 1 year, and developmental outcomes at 2 years.^31,32^

### Study oversight

Compliance to protocol and data integrity were maintained. Patient care data were stored and secured. Study recruitment criteria were reported to DSMB quarterly and IRB annually. Compliance measurements were documented as intake volumes, feeding durations, feeding positions, postprandial positions and symptom scores, growth metrics and nutritional status. Compliance to administration of omeprazole was confirmed using electronic medical records (Epic, Epic Systems Corporation, Verona, WI, USA) and or parental validation. Trial protocol and important changes to methods after trial commencement are listed in Supplement 1.

### Statistical methods

Based on our preliminary data, we had planned to enroll 100 patients (50 per group) to detect 27% or higher increase in proportion of success of the study group compared to the conventional group with 80% power and overall one-sided *α* level of 0.025. One interim futility analysis was planned at about 50% information prior to the final analysis at 100% information, corresponding to 50 and 100 evaluable patients, respectively. The boundary was determined using Lan-DeMets spending functions to simulate O’Brein−Fleming boundaries.^33^ Using the target proportion of success, the boundary at the futility analysis was *P* > 0.297.

**Fig. 1:**
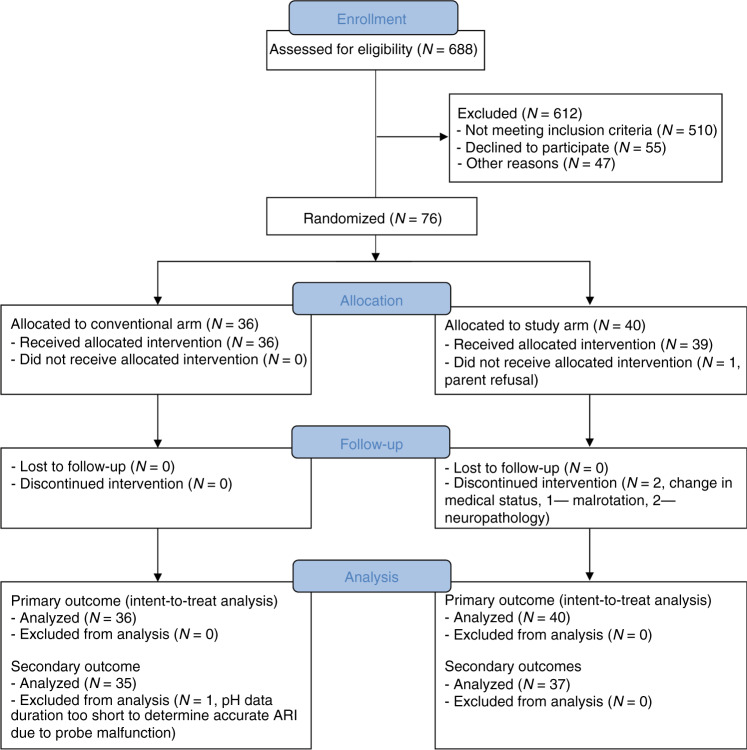
Study enrollment and randomization. Depicted is the CONSORT diagram describing participant flow and randomization into the conventional or study bundles, and subjects analyzed for outcomes.

Seventy-six infants were randomly assigned till the end of funding for this study and were included in the analysis of demographics and clinical characteristics (Fig. 1, Table 1) and primary outcome by intent-to-treat. If a patient dropped out before the end of study and no symptom score was evaluable, we treated the patient as a failure for the primary outcome by intention-to-treat. Secondary outcome analysis was performed for 72 subjects (Fig. 1). Futility boundary was not reached at interim analysis of 50 patients (*P* = 0.1 < 0.297) and accrual was continued with DSMB approval. Summary statistics were calculated for patient demographics and clinical characteristics for final analysis. Success rate in achieving PO or reduction in the I-GERQ-R by 6-points was calculated with 95% confidence interval and compared using chi-square test between the conventional and study groups (primary outcomes) for the intention-to-treat and treat-as-treated analyses. Fisher’s exact or chi-square test were used to compare other categorical secondary outcomes including feeding method and supplemental oxygen between the groups. Shapiro−Wilk test for normality was used for the continuous outcomes. Paired *t* tests or Wilcoxon signed-rank tests were used to assess changes in growth velocity and feeding therapy characteristics between time-1 and time-2. Two sample *t* tests or Wilcoxon rank-sum tests were used to compare these continuous outcomes between the conventional and study groups. Median (interquartile range (IQR)), mean (SD), or % was reported, unless stated otherwise. *P* values < 0.05 were considered statistically significant, and SAS version 9.4 (SAS, Inc, an IBM Company, Chicago, IL) was used.

**Table 1:** Baseline demographic and clinical characteristics of enrolled participants.

Variable	Overall subjects (*N* = 76)	Conventional group (*N* = 36)	Study group (*N* = 40)
At birth			
Gender, Female—*N* (%)	37 (49)	18 (50)	19 (48)
Race—*N* (%)			
African American	11 (15)	3 (8)	8 (20)
Asian	1 (1)	0 (0)	1 (3)
Other	6 (8)	2 (6)	4 (10)
Unknown	1 (1)	1 (3)	0 (0)
White	57 (75)	30 (83)	27 (68)
Ethnicity—*N* (%)			
Hispanic or Latino	2 (3)	2 (6)	0 (0)
Non-Hispanic or Latino	71 (93)	32 (89)	39 (98)
Unknown	3 (4)	2 (6)	1 (3)
Gestational age (GA)—wks	29.3 [27–32.9]	29.3 [28–32.2]	29.2 [27–33.1]
Preterm birth—*N* (%)	68 (90)	34 (94)	34 (85)
Birth weight—kg	1.23 [0.9–1.9]	1.2 [0.9–1.9]	1.3 [0.9–2.1]
Size for gestational age—*N* (%)			
Small (<10th %)	9 (12)	5 (14)	4 (10)
Average (10th−90th %)	58 (76)	26 (72)	32 (80)
Large (>90th %)	9 (12)	5 (14)	4 (10)
Cesarean delivery—*N* (%)	50 (66)	22 (61)	28 (70)
At inception			
Postmenstrual age—wks	41.1 (2.5)	41.3 (2.2)	40.9 (2.7)
Chronologic age—wks	10.9 (4.3)	11.1 (4.5)	10.7 (4.3)
Weight—kg	3.5 (0.8)	3.5 (0.8)	3.5 (0.8)
O_2_ requirement at 36 wks PMA—*N* (%)	36 (47)	17 (47)	19 (48)
O_2_ requirement at 28 days age—*N* (%)	46 (61)	21 (58)	25 (63)
Feeding method—*N* (%)			
Gavage	2 (3)	0 (0)	2 (5)
Transitional	37 (49)	18 (50)	19 (48)
Oral	37 (49)	18 (50)	19 (48)
Nasal cannula oxygen—*N* (%)	23 (30)	11 (31)	12 (30)
Total intake volume—mL/kg/day	150 [149–154]	150 [148–152]	150 [150–157]
Total oral intake volume—mL/kg/day	112 [45–150]	112 [57–150]	112 [22–146]
Acid reflux index (ARI)—%	9.3 [6.0–17.1]	10 [6.0–16.7]	9.1 [5.0–17.6]
ARI category—*N* (%)			
Indeterminate (ARI 3–7%)	26 (34)	12 (33)	14 (35)
Abnormal (ARI > 7%)	50 (66)	24 (67)	26 (65)

Data presented as *N* (%), median [IQR], or mean (SD).

## Results

### Participant characteristics

Screening, recruitment and follow-up of subjects occurred between August 2012 and October 2018, and data were locked till May 2019. Recruitment ended to allow for clinical outcome analysis. From the 688 infants assessed for eligibility, ARI was: normal (<3%) in 246 (36%), indeterminate (3–7%) in 169 (25%), and abnormal (>7%) in 273 (40%). Study enrollment, randomization, and primary outcome analysis are described in the CONSORT diagram (Fig. 1). Demographic and clinical characteristics at allocation were not significantly different in both groups (Table 1). Frequency (%) of GERD referral reasons were for respiratory concerns (apnea/bradycardia/desaturation, airway management, or suspected aspiration) in 54%, feeding concerns (poor oral feeding or intolerance) in 47%, and GERD-type symptoms (arching/irritability or emesis) in 25% (note proportions do not add to 100 due to providers being able to list multiple reasons for referral). Reasons for referral did not differ between the conventional and study groups (all *P* > 0.05). Proportion of milk types (exclusive breast milk:exclusive formula:combination of formula and breast milk, %) were not different between groups: 19:67:14 in the conventional vs. 18:53:3 in the study groups (*P* = 0.24). Of those formula-fed (28 in the conventional group and 29 in the study group), proportion of formula types (hydrolyzed:gentle:low lactose:preterm:standard, %) were: 4:7:4:75:10 in the conventional vs. 10:10:0:69:10 in the study groups (*P* = 0.84). Caloric density ranged from 19 to 30 cal/oz, and the proportions (%) (19:20:22:24:27:30 cal/oz) for the conventional (11: 31: 33: 19: 6) and the study groups (8:20:30:35:3) did not differ (*P* = 0.47). Breast milk intake in both groups was 40% at inception (*P* = 1.0), and the caloric density (cal/oz) for the conventional vs. study groups was 24 ^21–24^ and 24 ^21–26^ respectively (*P* = 0.41) For the conventional and study groups respectively, acid-suppressive dose (mg/kg/dose BID) was 0.75 [0.75–0.75] vs. 0.75 [0.75–0.75], *P* = 0.27 upon initial dose, and 0.75 [0.75–1.0] vs. 1.0 [0.75–1.0], *P* = 0.09 at follow-up.

### Study outcomes

#### Primary and secondary clinical outcomes

**Fig. 2:**
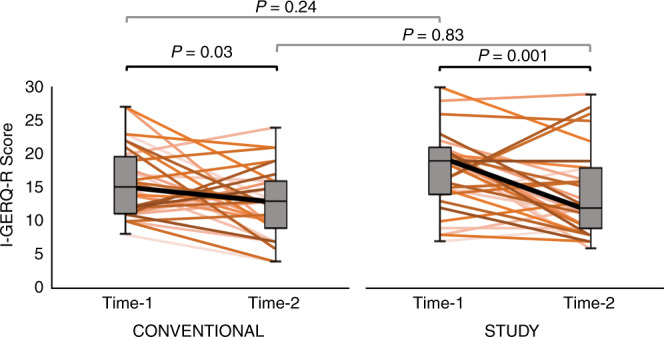
I-GERQ-R outcomes. Depicted is a combination plot by group (boxplots) and individual I-GERQ-R scores (black line represents median). Note that parent perception scores (I-GERQ-R) significantly decreased in both groups at Time-2.

The clinically meaningful primary and secondary outcomes did not differ significantly between the groups (Table 2). I-GERQ-R scores for the study and conventional groups are shown (Fig. 2). At inception: positive I-GERQ-R was 19/35 (54%) in the conventional group vs. 24/37 (65%) in the study group, *P* = 0.36. At Time-2: positive I-GERQ-R prevalence was 9/31 (29%) in the conventional group vs. 13/34 (38%) in the study group, *P* = 0.43. In the study group vs. conventional group, respectively: (a) primary outcome achieved in 33% (95% CI, 19−49%) vs. 44% (95% CI, 28−62%) (*P* = 0.28), (b) secondary outcomes: independent oral feeding in 65% (95% CI, 48−80%) vs. 77% (95% CI, 60−90%), *P* = 0.26, ≥6-point I-GERQ-R decrease in 38% (95% CI, 22−56%) vs. 35% (95% CI, 19−55%), *P* = 0.82, length of stay was 98 [81–132] days vs. 108 [83–125] days, *P* = 0.89, and oxygen requirement at discharge in 19% (95% CI, 8−35%) vs. 26% (95% CI, 13−43%), *P* = 0.49. There were no significant differences in growth metrics (all *P* > 0.05) or developmental scores at 2 years (all *P* > 0.05). Feeding outcomes or I-GERQ-R scores did not significantly differ between the conventional vs. study groups based on feeding method at inception (Table 3). Individual I-GERQ-R questions relating to vomiting, regurgitation, and crying (i.e. frequency of emesis, volume of emesis, symptoms with emesis, and crying more than usual in the past week) had no differences (*P* > 0.05) within the group or between the groups across maturation for these individual symptoms, except for symptoms with emesis (never:rarely:sometimes:often:always, %) was 16:16:42:19:6 for the conventional group at Time-1 vs. 18:18:29:0:35 for the study group at Time-1, *P* < 0.01.

**Table 2:** Primary and secondary clinical outcomes and compliance measures.

Primary outcome (intent-to-treat analysis)	Overall (*N* = 76)	Conventional (*N* = 36)	Study (*N* = 40)	*P* value
A priori clinical outcome, Success, *N* (%)	29 (38)	16 (44)	13 (33)	0.28

Secondary outcomes	Overall (*N* = 72)	Conventional (*N* = 35)	Study (*N* = 37)	*P* value
I-GERQ-R decrease by 6—*N* (%)	24/65 (37)	11/31 (35)	13/34 (38)	0.82
Feeding outcome at Time-2—*N* (%)				0.26
PO	51 (71)	27 (77)	24 (65)	
Transition (PO + Tube)	18 (25)	8 (23)	10 (27)	
Tube	3 (4)	0 (0)	3 (8)	
Length of hospital stay—days	108 [82–129]	108 [83–125]	98 [81–132]	0.89
Hospital stay inception to discharge—days	25 [11–45]	23 [3–39]	27 [16–47]	0.26
Weight growth velocity (GV)—g/day	27.1 (9.4)	26.5 (7.2)	27.6 (11.1)	0.64
Length GV—cm/day^a^	0.1 (0.1)	0.1 (0.1)	0.1 (0.1)	0.88
Head circumference GV—cm/day^a^	0.1 [0.0–0.1]	0.1 [0.0–0.1]	0.1 [0.0–0.1]	0.87
Feeding method at discharge—*N* (%)				0.32
PO	54 (75)	29 (83)	25 (68)	
Transition (PO + Tube)	14 (19)	5 (14)	9 (24)	
Tube	4 (6)	1 (3)	3 (8)	
Oxygen requirement at discharge—*N* (%)	16 (22)	9 (26)	7 (19)	0.49
Compliance to feeding methods				
Total Fluid Volume (TFV)				
TFV at inception—#	N/A	150 [147, 152]	150 [149, 156]	0.8
TFV at time-2—#	N/A	143 [134, 148]^a^	133 [126, 137]^a^	<0.001
# days TFV < 140/kg/d	N/A	10 [2, 26]	22 [11, 31]	0.005
TFV compliance (TFV < 140/kg/d for >75% of time)	N/A	9 (26)	27 (73)	<0.001
Position				
Feeding position, RSL, %	N/A	5 [1, 17]	78 [12, 94]	<0.001
Feeding in RSL > 75% of the time, %	N/A	0 (0)	21 (57)	<0.001
Supine position postprandial, %	N/A	50 [40, 66]	87 [74, 96]	<0.001
Supine position postprandial > 75% of the time, %	N/A	7/34 (21)	27/36 (75)	<0.001
Feeding duration				
% time feed duration >30 min, %	N/A	4 [0, 37]	15 [0, 69]	0.15
Feeding duration at inception, min	N/A	23 [17, 41]	30 [19, 34]	0.64
Feeding duration at Time-2, min	N/A	23 [19, 28]	26 [20, 30]	0.15
Long-term follow-up^b^				
Feeding method at 6 months—*N* (%)				0.65
PO	36/50 (72)	21/27 (78)	15/23 (65)	
Transition (PO + Tube)	10/50 (20)	4/27 (15)	6/23 (26)	
Tube	4/50 (8)	2/27 (7)	2/23 (9)	
Feeding method at 1 year—*N* (%)				0.11
PO	37/44 (84)	20/22 (91)	17/22 (77)	
Transition (PO + Tube)	4/44 (9)	0/22 (0)	4/22 (18)	
Tube	3/44 (7)	2/22 (9)	1/22 (5)	
CCA BSID-III				
Cognitive Score <80—*N* (%)	9/44 (21)	5/23 (22)	4/21 (19)	1.00
Cognitive Score—#	95 [90–105]	100 [90–105]	90 [90–100]	0.11
Receptive Communication <80—*N* (%)	14/42 (33)	8/23 (35)	6/19 (32)	0.83
Receptive Communication Score—#	90 [77–103]	91 [74–103]	89 [77–103]	0.75
Expressive Communication <80—*N* (%)	11/25 (44)	7/14 (50)	4/11 (36)	0.69
Expressive Communication Score—#	86 [71–100]	81 [71–94]	89 [71–103]	0.66
Fine Motor <80—*N* (%)	11/41 (27)	5/23 (22)	6/18 (33)	0.49
Fine Motor Score—#	94 [79–103]	97 [82–110]	88 [70–97]	0.13

Data presented as *N* (%), median [IQR], or mean (SD).

^a^One value missing from both conventional and study groups.

^b^Data were not available for all subjects for long-term follow-up outcomes, *N* values are reported.

**Table 3:** Feeding and I-GERQ-R outcomes by feeding method at inception.

Outcome	Conventional	Study	*P* value
Among those tube-fed at inception	*N* = 18	*N* = 20	
Achieved exclusive oral feeding	11 (61)	7 (35)	0.11
Achieved I-GERQ-R decrease ≥ 6	7/17 (41)	8 (40)	0.94
Achieved PO or I-GERQ-R decrease ≥ 6	14 (78)	13 (65)	0.39
Achieved exclusive oral feeding + I-GERQ-R decrease ≥ 6	4/17 (24)	2 (10)	0.38
Among those PO-fed at inception	*N* = 17	*N* = 17	
Maintained PO	16 (94)	17 (100)	1.0
Achieved I-GERQ-R decrease ≥ 6	4/14 (29)	5/14 (36)	1.0
Maintained PO or I-GERQ-R decrease ≥ 6	16 (94)	17 (100)	1.0
Maintained PO + I-GERQ-R decrease ≥ 6	4/14 (29)	5/14 (36)	1.0

Data presented as *N* (%).

#### Compliance measures, side effects and adverse events

Compliance to randomization, allocation and interventions, and drop outs are reported (Fig. 1, Table 2). Total fluid volume was identical at inception but both groups showed a reduction compared to baseline at Time-2. However, as per the trial design, the study group showed significantly (all *P* < 0.05) lower volume intake, feeding in right side lying position, and postprandial supine position. Feeding duration of actual feeding was increased in the study group but not statistically different from the conventional group. No side effects or adverse events were reported in either group.

## Discussion

In this RCT, while controlling for birth gestation and severity of acidity, we compared the effectiveness of acid suppression with or without a systematic feeding modification bundle in modifying feeding outcomes and I-GERQ-R scores. We found no differences in our a priori primary outcome or preassigned secondary outcomes. Important clinical and research implications can be noted despite the nonsuperiority of the feeding bundle.

Diagnostic conundrums and management issues with GERD in the NICU setting persist. Prior studies^8,18,19^ used perceived clinical symptoms as a basis for acid suppression, but studies have shown lack of benefit on symptom improvement.^34,35^ Recent work by us^6,20,21^ suggests that such symptoms are often due to pharyngo-esophageal provocation or cross-systems activation of reflexes, and can occur during nonacid events or swallowing events, or during transient relaxation of lower esophageal sphincter.^29^ However, the inability to handle the refluxate determines the “troublesomeness of the symptoms” rather than the esophageal acid exposure. Clinical practice varies when pathophysiological reasoning is not commonly applied. Clinical practices can have unintended consequences^1,36–39^ resulting from acid suppression, undernutrition, delays with feeding milestones, decisional conflicts, discharge outcomes and prolonged hospitalization, all of which can escalate burden.^9,26^

Salient features of our study include (1) allocations were unbiased and appropriately distributed between the groups; (2) among those presenting with GERD symptoms at inception, about 36% of infants had normal esophageal acid exposure, while 40% had abnormal acid exposure, and the rest in the indeterminate range; (3) the study bundle was not superior to acid suppression alone in improving primary outcomes or secondary outcomes; (4) restricted feeding volume, body positions (intra- and postprandial), oral or gavage feeding methods, supplemental oxygen, birth gestation and postnatal maturation did not influence the primary or secondary outcomes; (5) reliability of compliance among those discharged was based on parental trust and available information; (6) there were no reported adverse events. No differences in long-term developmental outcomes or economic burden measures, such as, LOHS, feeding methods and respiratory support at discharge were noted; (7) symptom scores (I-GERQ-R) were significantly lower in both groups, suggesting that maturation may play a role in symptom modification, and not the bundled approaches; (8) feeding outcomes improved in both the groups.

GMT trial strengths and clinical implications are several: (1) Random allocation, study design and protocol adherence were robust and rigorous. Although our strict inclusion criteria may have led to lower eligibility, our approach resulted in identifying infants carefully with true ARI as a marker of esophageal acid exposure. Objective determination of GERD based on ARI > 7% is justifiable in future trials, as nearly 40% of infants are in this severe range, and it is possible to study such a group in larger clinical trials based on pH and impedance criteria, while employing placebo for equipoise. Since time-limited PPI therapy concurrent with feeding strategies was neither shown to be beneficial or associated with adverse effects, we believe that it is safe to include a completely untreated placebo group in future trials that enroll patients with objectively determined acid-GERD. (2) The management strategies were tightly regulated, as were feeding and testing guidelines, and the treatment was uniformly delivered across the two groups. The patient population was homogeneous and constituted a fair representation from the convalescing NICU population. In addition, the randomized controlled allocation accounted for premature or full-term birth, and the indeterminate or determinate acid-GERD per ARI. Furthermore, the prevalence of oxygen requirement or tube feeding at discharge was not different between the groups. (3) Our study trial has many elements of objectivity. Determination of I-GERQ-R and ARI, as well as monitoring feeding methods during the trial are strengths. Thirty-six percent of those with aerodigestive and or cardiorespiratory symptoms perceived by their clinicians to be due to GERD prior to trial consent were not randomized and were also never treated with a PPI as the esophageal acid exposure was normal (ARI < 3%). In a purely symptom-based clinical trial, all those 688 infants screened would have likely been treated for presumed GERD. In the current study, only those that had true ARI exposure have been randomized and treated. Therefore, the symptom-based approach alone is not the solution to diagnose and treat GERD. Interestingly, perception of symptoms (IGERQR scores) decreased across time regardless of treatment group allocations (Fig. 2). These findings strongly support maturational effect. As both the groups were treated with PPI, placebo-included RCTs are needed to determine if maturation alone will improve objectively determined acid- and nonacid-GERD. (4) Absence of pH-impedance testing to confirm true acid-GERD prior to randomization would have resulted in all patients being treated based on subjective, nonspecific symptoms alone. Owing to the strict inclusion and exclusion criteria of this RCT, those with indeterminate and abnormal ARI were treated with a PPI. In the future, a careful RCT that tests the utility of PPI treatment for confirmed acid-GERD by allocating patients to either limited PPI treatment or placebo is indicated to determine whether PPI treatment is needed. The effectiveness of our short-term use of PPI for 4 weeks to improve GERD-attributable symptoms should be tested in future trials. Effect of esophageal acid exposure and therapies on primary mechanistic outcomes of esophageal motility and symptom causation will be addressed in future reports. (5) In routine clinical practice, feeding volumes are modified and alterations in feeding positions are used to manage symptoms. Our study did not show any differences in the outcomes with feeding- and position modifications. Furthermore, volume restriction had no influence on the study outcomes. The improvement in symptoms and feeding outcomes over time irrespective of PPI or feeding modifications may suggest a maturational effect.

It is important to note that major mechanisms of GER, i.e. transient LES relaxation is the major reason for any reflux events—both acid or nonacid substrate. Our therapeutic target was ARI in this study via PPI, feeding volumes, and positional changes. Given that acid GER can also have weakly or nonacid either before or after PPI therapy, and that there were no differences in outcomes between the two groups, we speculate that neither PPI, feeding volume, or positional changes modify the studied indices or symptoms. Maturation under optimal conditions of good nutrition along with placebo-controlled trials are needed to answer the importance of weakly acid or nonacid GER, which would require a multicenter trial with a large group of infants with appropriate physiologic diagnostic testing.

Our study has limitations: (1) Parental and physician biases appeared to be a barrier to recruitment. Recruitment was slow despite the high prevalence of GERD-associated symptoms and a high eligibility rate. This is concerning, as many parents refused clinical trial participation. Many infants did not have true acid-GERD, and fluid restriction often occurred before testing. Interestingly, in some cases, parents and providers did not want to stop the PPI use. These barriers to recruitment can be mitigated in future larger trials with better parent−provider education, as no major effects on the primary or secondary outcomes were noted in our study with or without our allocated bundled GERD treatment. (2) Owing to the higher screening to eligibility ratio, rigorous inclusion criteria, and strict study protocols, we could not complete the recruitment as originally planned of 100 evaluable patients. Seventy-six infants were randomly assigned during the funding period. However, using this cumulative sample size of 72 patients for interim monitoring, we found that we would stop for futility at this time point even if the funding period was not ended. (3) Further studies are needed to correlate parental/provider perception of symptoms (I-GERQ-R) with true symptoms and symptom indices examined during pH-impedance testing. Such studies should also address the severity of acid exposure index in relation to changes in symptom indices.

## Conclusion

We addressed the current practice controversies in this clinical trial: (1) Screening and identifying acid-GERD objectively is possible in symptomatic infants prior to any pharmacotherapy. (2) Feeding strategy modification (fluid restriction, positional changes, prolonged feeding duration) has no role in decreasing reflux-type symptoms or in improving the primary outcome of achieving independent oral feeds and/or a 6-point decrease in I-GERQ-R score. (3) No difference in the prevalence of chronic lung disease was noted between the groups. (4) I-GERQ-R scores decreased across time regardless of treatment group allocations that strongly support maturational effect. However, we did not detect an effect on a priori short- or long-term outcomes following randomized allocations. As restrictive feeding strategies do not make a difference, placebo-controlled clinical trials in a larger cohort of convalescing NICU infants with objectively determined newer GERD criteria must be addressed in future trials.

## Supplementary information

Supplementary Information
