# Supplementary material for: Role of feeding strategy bundle with acid-suppressive therapy in infants with esophageal acid reflux exposure: a randomized controlled trial
Source: Pediatr Res. 2020 May 7;89(3):645–52. doi: 10.1038/s41390-020-0932-4 (PMC7647955; doi:10.1038/s41390-020-0932-4)
Supplement: Supplementary file 1 — Supplementary Information [file 41390_2020_932_MOESM1_ESM.docx]

**PROTOCOL TITLE:**

Pathophysiology of the Aerodigestive Reflex in Infants: GERD Management and Therapy Trial [GMT Trial]

**PRINCIPAL INVESTIGATOR:**

Name: Sudarshan Jadcherla, MD, FAAP, FRCP (Ireland) AGAF

Department/Center: Center for Perinatal Research

Telephone Number: (614) 655-6643

Email Address: Sudarshan.Jadcherla@nationwidechildrens.org

**VERSION NUMBER/DATE:**

Version 9.0 6/6/2019

**Nationwide Children’s Hospital IRB REVISION HISTORY**

| **Revision #** | **Version Date** | **Summary of Changes** | **Consent Change?** |
| --- | --- | --- | --- |
| 1 | 11/30/2012 | Because NICU infants have lower fluid prescriptions in general, revised allowable total fluid volume from ≥170 ml/kg/day to ≥150 ml/kg/day at inception to be eligible for screening | No |
| 2 | 2/15/2013 | Because convalescing NICU infants with lung disease can have varying supplemental oxygen requirements, revised allowable respiratory support from Room air or ≤0.3 LPM via nasal cannula to ≤1.0 LPM or Room air at inception to be eligible for screening | No |
| 3 | 12/9/2013 | Because parents and providers did not permit stopping the PPI at 4 weeks for clinical reasons, allowed the subject to remain on medication at the follow up evaluation | No |
| 4 | 3/24/2014 | Allowed Practitioners to increase permissible study medication dose as clinically indicated | No |
| 5 | 7/29/14 | Added the use of swallowing motility measurements to the mechanistic study protocol | Yes |
| 6 | 9/19/2014 | Convalescing subjects with Neonatal Abstinence syndrome admitted to NICU have feeding difficulties masquerading as GERD symptoms. Changed inclusion criteria to permit these infants to be screened for eligibility into the study | No |
| 7 | 5/1/2015 | To examine sucking, swallowing and motility measures, sucking-swallowing rhythms were measured for the mechanistic study protocol | Yes |
| 8 | 1/12/2018 | Added reciprocity with The Ohio State University and included biostatistician, Wei Lai into the study staff | yes |

# Study Summary

| **Study Title** | Pathophysiology of Aerodigestive Reflex in Infants: GERD Management Trial |
| --- | --- |
| **Study Design** | Single center randomized control trial |
| **Primary Objective** | To compare two feeding approaches and their effect on clinical outcomes in infants with proven Gastroesophageal Reflux Disease (GERD) |
| **Secondary Objective(s)** | To determine the pathophysiological mechanism of success or failure to either therapy, we will test the hypothesis and validate results from the preliminary data by utilizing our diagnostic tools to identify differences between the two study arms in regards to: aerodigestive reflexes, esophageal clearance mechanisms, neuromotor markers of swallowing, and pH-Impedance-symptom indices. |
| **Research Intervention(s)/ Investigational Agent(s)** | Both groups will have esophageal manometry studies followed by randomization into two groups, Conventional and Study. The **conventional group** allows for unrestricted feeding volumes with no time limit, feeds given in any positon and random post prandial feeding positions. **The study group** will have permissive feeding volume restrictions, feeding duration of at least 30 minutes, feeding given in right side lying, and supine post prandial position. Both groups will be treated with omeprazole per NCH NICU guidelines |
| **IND/IDE #** | N/A |
| **Study Population** | Infants admitted to the NICU at NCH and referred to the Neonatal and Infant Feeding Disorders program for evaluation of Gastroesophageal reflux Disease (GERD) |
| **Sample Size** | 120 |
| **Study Duration for individual participants** | 5 weeks |
| **Study Specific Abbreviations/ Definitions** | GMT (GERD Management and Therapy Trial)  RSL- right side lying  ARI –Acid reflux index  PMA= (Gestational age at birth + chronologic age) |

# Objectives

**2.1 Purpose, specific aims or objectives**

- Clinical trial based on objective evaluation of symptoms scored by blinded independent rater during pH-Impedance studies (Aim 1).
- Investigates the neuro-physiological aspects of the reflexes involved with GERD and its complications (Aim 2).
- Unique to this proposal, we will apply physiologically rational, and safe, therapeutic strategies that have the real potential to ameliorate GERD symptoms/complications. If the proposed aims are achieved, we anticipate:
- Finding that not only acid, but also feeding volume, position and maturational changes in adaptation underlie the pathogenesis of GERD, and in this way clearly identifying relevant new therapeutic targets for this disease.
- Understanding the various stimulus-targets and provoked reflexes that lead to sensitization of afferents, and thereby symptoms associated with GERD.
- Understanding the mechanism(s) underlying functional esophageal disorders in infants, thereby clarifying the pathophysiology of symptoms presumed to be GERD related.
- Development of cost effective, efficacious methods for the diagnosis and management of GERD in infants.
- Establishing objective criteria for evaluating the need for anti-reflux surgical procedures in infants.

**2.2 Hypothesis**

- Aim 1
  - The innovative feeding strategy provided over 4 wks is more effective than standard therapy for the acquisition of safe feeding skills by improved symptom scores (Primary), improved individual I-GERQ-R measures, growth measures, reduced lengths of stay and resource utilization (Secondary).
- Aim 2
  - The sensory-motor characteristics of aerodigestive reflexes evoked with induced esophageal stimulation or upon spontaneous esophageal provocation as in GER, improve in the innovative therapy group with regards to: sensory thresholds, response latency and duration, frequency and magnitude of reflexes, TLESRs, spatial-temporal-physical- chemical characteristics of GER events with associated symptom indices.

# Background

**3.1 Relevant prior experience and gaps in knowledge**

- The NASPGHAN-2009 guidelines define GERD as reflux of gastric contents in the presence of troublesome symptoms and/or complications. The prevalence of GERD in premature infants is estimated to be 10.6 %. Premature infants have pathologic reflux with esophageal manifestations (e.g. irritability, feeding refusal, arching, dysphagia) and extra- esophageal manifestations (e.g. arousals, cardiorespiratory-, sensory-, and physical symptoms, chronic lung disease, impaired growth). Such high risk infants suffer chronic morbidity, longer hospitalizations, re- admissions, and account for an unacceptable health care burden.
- Current treatment approaches in infants are likely to fail because of lack of clear therapeutic targets. Furthermore, GERD symptoms are non-specific and heterogeneous such that designing effective approaches to symptom based management is very difficult.
- Exact economic burden of premature infant GERD is lacking. 12.7% (525,000) out of 4,131,019 births in 2009 were born at < 37 wks. Assuming that half (263,000) of these infants are in NICUs, and given our estimated prevalence of GERD as 10.6% (27,900) and the estimated cost per admission attributable to GERD was $76,796; we project an estimated economic burden attributable to GERD as over $2.1 billion. Others have estimated that 48% of ICU discharges are treated for presumed GERD. Clinical significance of GERD is also evident from the 7-fold increase in the use of acid suppressive medications, and indeed about 45% of anti-reflux procedures are performed in infants (15). These numbers ignore the quality of life issues for both the patients and the parents.
- Extensive developments with the mechanisms, physiology, pharmacotherapy and clinical approach to GERD in the human adult, as well as therapeutic limitations in infants are well recognized Specifically for infants, there are no available safe or FDA-approved prokinetics that regulate esophageal motility and suppress transient lower esophageal sphincter (LES) relaxations.
  1. **Relevant Preliminary Data**
- In an observational study in infants (N=35) referred for GERD management, we studied the impact of feeding methods (volumes, duration, flow rates, caloric density, osmolality) on pH-Impedance variables of GER. The conclusions were: longer feeding duration, less feeding volume, and slower flow rate led to less total GER events, fewer nonacid GER events and shorter bolus clearance time; whereas, caloric density and osmolality of feeds has no impact on the characteristics of GER.
  - Jadcherla SR, Chan CY, Moore R, Malkar M, Timan CJ, Valentine CJ 2011 Impact of Feeding Strategies on the Frequency and Clearance of Acid and Nonacid Gastroesophageal Reflux events in dysphagic Neonates. JPEN J Parenter Enteral Nutr.
  - Malkar M, Chan CY, Peng J, Moore R, Jadcherla SR 2011 Effect of osmolality and caloric density of feeds on the frequency and characteristics of gastroesophageal reflux in infants. Neurogastroenterol Motil.
- The effect of right lateral position on accelerated gastric emptying has been described by others (107, 108, 114), and the effect of supine posture in the prevention of ALTEs or SIDS is recognized in the AAP guidelines.
  - 2003 Apnea, sudden infant death syndrome, and home monitoring. Pediatrics 111:914-917.
  - Omari TI, Rommel N, Staunton E, Lontis R, Goodchild L, Haslam RR, Dent J, Davidson GP 2004. Paradoxical impact of body positioning on gastroesophageal reflux and gastric emptying in the premature neonate. J Pediatr 145:194-200.
  - Corvaglia L, Rotatori R, Ferlini M, Aceti A, Ancora G, Faldella G 2007 The effect of body positioning on gastroesophageal reflux in premature infants: evaluation by combined impedance and pH monitoring. J Pediatr 151:591-596, 596 e591.
  - Van Wijk MP, Benninga MA, Dent J, Lontis R, Goodchild L, McCall LM, Haslam R, Davidson GP, Omari T 2007 Effect of body position changes on postprandial gastroesophageal reflux and gastric emptying in the healthy premature neonate. J Pediatr 151:585-590, 590 e581-582.
- We used state-of-the-art methods including, multimodal esophageal sensory provocation concurrent with video, pH-Impedance and symptom indices in the investigation of sensory-motor aspects (sensory thresholds, reflex frequency, response latency, duration and magnitude) of Vago-vagal reflexes (Primary and Secondary Peristalsis, Upper esophageal sphincter contractile reflex-UESCR, Lower esophageal sphincter relaxation reflex-LESRR and Pharyngeal reflexive swallowing) that facilitate swallowing and esophageal clearance to maintain airway protection in neonates. Twelve pharyngo esophageal motility studies were done in 6 subjects, 3 in each group, at inception (test-1) and again at completion of therapy (test-2). With innovative strategy, the response onset to esophageal stimulation induced LESRR is decreased at T2 in addition to a greater LES relaxation magnitude compared to T1; in contrast, response onset to LESRR and LESRR magnitude are similar at T1 and T2 for standard therapy. This finding supports the hypothesis that LES relaxation governed by inhibitory vagal effects mediated by nitrergic neurons is rapidly restored with innovative feeding strategy, similar to those changes tested during maturation.
  - Pena EM, Parks VN, Peng J, Fernandez SA, Di Lorenzo C, Shaker R, Jadcherla SR 2010 Lower esophageal sphincter relaxation reflex kinetics: effects of peristaltic reflexes and maturation in human premature neonates. Am J Physiol Gastrointest Liver Physiol 299:G1386-1395.
- Clinical outcomes from 44 subjects that had reflux index >3% were measured; 24 infants had lower feeding volumes, longer feeding duration, greater compliance to supine postprandial posture (innovative feeding strategy); 20 infants had standard feeding therapy, all received acid suppressive therapy. This trial supported the hypothesis that the innovative feeding strategy is superior to standard feeding therapy.
  1. **Statistical procedures**
- Statistical Analyses:
  - Aim I: Outcomes: We will test the hypothesis that the innovative feeding strategy provided over 4 wks is more effective than standard therapy for the acquisition of safe feeding skills by improved symptom scores (Primary), improved individual I-GERQ-R measures, growth measures, reduced lengths of stay and resource utilization (Secondary). Linear mixed models will be used to study the associations between the primary endpoints for Aim 1 (symptom scores) and treatment, and trend across time. The interaction term “group x time” will be included in the model to study whether the two treatment groups have different “behavior” across time. Confounder variables, such as medications, feeding position or volume, will be included in the models. Logistic regression models will be used to assess success of the treatment vs. standard of care. Also, potential confounders (e.g. feeding method: oral vs. tube) will be included and studied in these models. Holm’s method will be used to adjust for multiple comparisons (150). All the secondary variables listed above, will be analyzed using either linear mixed models or logistic regression models. Model goodness of fit tests and residual analyses will be performed to assess the models. Interaction terms and potential confounder variables will be included.
  - Aim II: Mechanistic Hypothesis: We will test the hypothesis that the sensory-motor characteristics of aerodigestive reflexes evoked with induced esophageal stimulation or upon spontaneous esophageal provocation as in GER, improve in the innovative therapy group with regards to: sensory thresholds, response latency and duration, frequency and magnitude of reflexes, TLESRs, spatial-temporal-physical- chemical characteristics of GER events with associated symptom indices. Based on the preliminary data, we will have at least 80% power to detect the above differences of pre and post therapy between the two groups. Linear mixed models or logistic regression models (depending on the nature of the outcome variable) will be used to study the associations of interest within sensory motor components of aerodigestive reflexes within and between study groups and their outcomes. Potential confounders as well as interaction between groups and type of infusion will be included in the models. We have utilized similar models previously. Correlation between symptom indices (SI, SSI, SAP) and reflexes will be calculated.
- Sample Size Determination:
  - From the more recent preliminary data analysis, 83.3% (20/24, 95% CI: 62.6%-95.2%) of patients had success based on symptoms and feeding methods in the innovative strategy group, while only 35% (7/20, 95% CI: 15.4%-59.2%) had success in the standard therapy group. In order to provide greater assurance than afforded by comparing to historical control of the standard therapy, a total of evaluable 100 patients will be randomized into the innovative feeding strategy or standard therapy group. The chi- square test yields 80% power to detect 27% or higher increase in proportion of success while maintaining an overall one-sided α level of 0.025. This conservative power calculation is supported by the preliminary data and assumes the proportion of success for the standard therapy is around 40% and a group-sequential design with O’Brien-Fleming error spending function.
- Interim Analysis:
  - One interim futility analysis will be undertaken at about 50% information prior to the final analysis at 100% information, corresponding to 50 and 100 evaluable patients, respectively. The boundary is determined using Lan-DeMets spending functions to simulate O’Brien-Fleming boundaries with an overall one-sided α level of 0.025. Using the target proportion of success, the boundary at the futility analysis expressed as a p-value is 0.297. We plan to enroll 120 patients to allow for 20% of attrition.
- Intention to treat analysis:
  - Every patient who had been randomized will be included in the analysis. If a patient drops off before end of study and no symptom score has been measured, we will treat the patient as a failure. Beside the intention to treat analysis, we will also perform the analysis with only those patients that complete the study. Linear mixed models will be used to study the associations between the primary endpoints for Aim 1 (symptom scores) and treatment, and trend across time. The interaction term “group x time” will be included in the model to study whether the two treatment groups have different “behavior” across time. Confounder variables, such as medications, feeding position or volume, will be included in the models. Logistic regression models will be used to assess success of the treatment vs. standard of care. Also, potential confounders (e.g. feeding method: oral vs. tube) will be included and studied in these models. Holm’s method will be used to adjust for multiple comparisons. All the secondary variables listed above, will be analyzed using either linear mixed models or logistic regression models. Model goodness of fit tests and residual analyses will be performed to assess the models. Interaction terms and potential confounder variables will be included.
  - The proposed projects will focus on esophageal provoking stimuli in designing multi-faceted innovative therapies, that will be studied in a prospective single center randomized blinded controlled trial. The pH- Impedance data with associated symptom indices will be analyzed. This trial will advance our knowledge of GER and GERD in infants. These studies will likely mandate the development of infant-specific diagnostic and treatment paradigms to manage not only GERD but also other diseases where GERD is a co-morbidity.

**3.4 Scientific or scholarly background for, rationale for, and signficance of the research based on the existing literature and how will it add to existing knowledge**

- This proposal is a logical extension of our current R01 and is built on Novel Concepts, and State-Of-The-Art Methods. Previously we have developed, validated, and safely used the approaches and equipment necessary to assess GER and GERD in infants including: a) novel multimodal esophageal sensory-motor testing using manometry provocation methods, b) respiratory inductance plethysmography, c) ultrasonography of glottal motion, d) video-manometry, e) pH-Impedance with direct observation/video correlation of symptoms, and f) robust analytical and statistical paradigms. We are translating these methods to improve outcomes in infants with feeding problems, e.g., improving successful feeding outcomes and avoidance of gastrostomy in those referred for G-tube placement.
- We will explore the sensory-motor mechanisms leading to symptoms in infants with GERD. GERD symptoms can be attributed to: a) refluxate properties: physical (gas, mixed, liquid), chemical (acid, non-acid), spatial (high or low) or temporal (acid or bolus clearance time); or b) luminal clearance mechanisms: esophageal mechano-distention and chemo-sensitive stimulation induced aerodigestive reflexes. The mechanisms for symptoms may underlie in stimulus thresholds, response-frequency, response-latency and response-magnitude of the aerodigestive protective reflexes. Therefore, to test the effects of different treatment strategies on these aerodigestive reflexes, we will interrogate the sensory-motor components before and after proposed therapies.
- Publication history and references pertinent to this proposal are as follows:
  - Vandenplas Y, Rudolph CD, Di Lorenzo C, Hassall E, Liptak G, Mazur L, Sondheimer J, Staiano A, Thomson M, Veereman-Wauters G, Wenzl TG, North American Society for Pediatric Gastroenterology H, Nutrition, European Society for Pediatric Gastroenterology H 2009 Pediatric gastroesophageal reflux clinical practice guidelines: joint recommendations of the North American Society for Pediatric Gastroenterology, Hepatology, and Nutrition (NASPGHAN) and the European Society for Pediatric Gastroenterology, Hepatology, and Nutrition (ESPGHAN). J Pediatr Gastroenterol Nutr 49:498-547.
  - **Jadcherla SR**, Chisolm D, Gardner W 2011 Economic burden of gastroesophageal reflux disease (GERD) in convalescing ICU Neonates. Neurogastroenterol Motil. (Accepted abstract)
  - **Jadcherla SR**, Peng J, Moore R, Saavedra J, Shepherd E, Fernandez S, Erdman SH, Di Lorenzo C 2011 Impact of personalized feeding program in 100 NICU infants: A novel pathophysiology-based approach for better outcomes. J Pediatr Gastroenterol Nutr. (In press)
  - **Jadcherla SR**, Chan CY, Moore R, Malkar M, Timan CJ, Valentine CJ 2011 Impact of feeding Strategies on the Frequency and Clearance of Acid and Nonacid Gastroesophageal Reflux events in dysphagic Neonates. JPEN J Parenter Enteral Nutr. (In press)
  - Malkar M, Chan CY, Peng J, Moore R, **Jadcherla SR** 2011 Effect of osmolality and caloric density of feeds on the frequency and characteristics of gastroesophageal reflux in infants. Neurogastroenterol Motil. (Accepted abstract)
  - 1976 Commentary on breast-feeding and infant formulas, including proposed standards for formulas. Pediatrics 57:278-285.
  - Kleinman RE 2009 Pediatric Nutrition Handbook 6th ed. Elk Grove Village, IL: American Academy Of Pediatrics Committee on Nutrition.
  - **Jadcherla SR**, Gupta A, Fernandez S, Nelin LD, Castile R, Gest AL, Welty S 2008 Spatiotemporal characteristics of acid refluxate and relationship to symptoms in premature and term infants with chronic lung disease. Am J Gastroenterol 103:720-728.
  - **Jadcherla SR**, Peng J, Chan CY, Moore R, Wei L, Fernandez S, Di Lorenzo C 2011 Significance of Gastroesophageal Refluxate in Relation to Physical, Chemical and Spatio-temporal Characteristics in Symptomatic ICU Neonates. Pediatr Res.(In press; PMID:21505374)
  - Behrman RE, Butler AS 2007 Preterm birth: causes, consequences, and prevention. The National Academies of Sciences, Institute of Medicine, Washington DC, p 740.2008 Hospital discharge of the high-risk neonate. Pediatrics 122:1119-1126.
  - Clark RH, Bloom BT, Spitzer AR, Gerstmann DR 2006 Reported medication use in the neonatal intensive care unit: data from a large national data set. Pediatrics 117:1979-1987.
  - **Jadcherla SR**, Wang M, Vijayapal AS, Leuthner SR 2010 Impact of prematurity and co-morbidities on feeding milestones in neonates: a retrospective study. J Perinatol 30:201-208.
  - Lasser MS, Liao JG, Burd RS 2006 National trends in the use of antireflux procedures for children. Pediatrics 118:1828-1835.
  - Malcolm WF, Gantz M, Martin RJ, Goldstein RF, Goldberg RN, Cotten CM 2008 Use of medications for gastroesophageal reflux at discharge among extremely low birth weight infants. Pediatrics 121:22-27.
  - Gupta A, **Jadcherla SR** 2006 The relationship between somatic growth and in vivo esophageal segmental and sphincteric growth in human neonates. J Pediatr Gastroenterol Nutr 43:35-41.
  - **Jadcherla SR** 2006 Esophageal Motility in the Human Neonate. NeoReviews 7:e7-e12.
  - Jadcherla SR 2006 Upstream effect of esophageal distention: effect on airway. Curr Gastroenterol Rep 8:190-194.
  - **Jadcherla SR**, Gupta A, Stoner E, Coley BD, Wiet GJ, Shaker R 2006 Correlation of glottal closure using concurrent ultrasonography and nasolaryngoscopy in children: a novel approach to evaluate glottal status. Dysphagia 21:75-81.
  - **Jadcherla SR**, Hoffmann RG, Shaker R 2006 Effect of maturation of the magnitude of mechanosensitive and chemosensitive reflexes in the premature human esophagus. J Pediatr 149:77-82.
  - **Jadcherla SR**, Gupta A, Coley BD, Fernandez S, Shaker R 2007 Esophago-glottal closure reflex in human infants: a novel reflex elicited with concurrent manometry and ultrasonography. Am J Gastroenterol 102:2286-2293.
  - **Jadcherla SR**, Gupta A, Stoner E, Fernandez S, Shaker R 2007 Pharyngeal swallowing: defining pharyngeal and upper esophageal sphincter relationships in human neonates. J Pediatr 151:597-603.
  - **Jadcherla SR**, Gupta A, Stoner E, Fernandez S, Caniano D, Rudolph CD 2008 Neuromotor markers of esophageal motility in feeding intolerant infants with gastroschisis. J Pediatr Gastroenterol Nutr 47:158-164.
  - Gupta A, Gulati P, Kim W, Fernandez S, Shaker R, **Jadcherla SR** 2009 Effect of postnatal maturation on the mechanisms of esophageal propulsion in preterm human neonates: primary and secondary peristalsis. Am J Gastroenterol 104:411-419.
  - **Jadcherla SR**, Gupta A, Wang M, Coley BD, Fernandez S, Shaker R 2009 Definition and implications of novel pharyngo-glottal reflex in human infants using concurrent manometry ultrasonography. Am J Gastroenterol 104:2572-2582.
  - **Jadcherla SR**, Stoner E, Gupta A, Bates DG, Fernandez S, Di Lorenzo C, Linscheid T 2009 Evaluation and management of neonatal dysphagia: impact of pharyngoesophageal motility studies and multidisciplinary feeding strategy. J Pediatr Gastroenterol Nutr 48:186-192.
  - **Jadcherla SR**, Vijayapal AS, Leuthner S 2009 Feeding abilities in neonates with congenital heart disease: a retrospective study. J Perinatol 29:112-118.
  - **Jadcherla SR**, Hogan WJ, Shaker R 2010 Physiology and pathophysiology of glottic reflexes and pulmonary aspiration: from neonates to adults. Semin Respir Crit Care Med 31:554-560.
  - Pena EM, Parks VN, Peng J, Fernandez SA, Di Lorenzo C, Shaker R, **Jadcherla SR** 2010 Lower esophageal sphincter relaxation reflex kinetics: effects of peristaltic reflexes and maturation in human premature neonates. Am J Physiol Gastrointest Liver Physiol 299:G1386-1395.
  - **Jadcherla SR** 2011 Development of esophageal peristaltic and defensive functions in infants: A synopsis. New York Academy of Sciences
  - **Jadcherla SR**, Duong HQ, Hofmann C, Hoffmann R, Shaker R 2005 Characteristics of upper oesophageal sphincter and oesophageal body during maturation in healthy human neonates compared with adults. Neurogastroenterol Motil 17:663-670.
  - **Jadcherla SR**, Rudolph CD 2005 Gastroesophageal Reflux in the Preterm Neonate NeoReviews 6:e87-e98.
  - Kleinman L, Rothman M, Strauss R, Orenstein SR, Nelson S, Vandenplas Y, Cucchiara S, Revicki DA 2006 The infant gastroesophageal reflux questionnaire revised: development and validation as an evaluative instrument. Clin Gastroenterol Hepatol 4:588-596.
  - Nelson SP, Chen EH, Syniar GM, Christoffel KK 1997 Prevalence of symptoms of gastroesophageal reflux during infancy. A pediatric practice-based survey. Pediatric Practice Research Group. Arch Pediatr Adolesc Med 151:569-572.
  - Orenstein SR 1997 Infantile reflux: different from adult reflux. Am J Med 103:114S-119S.
  - Orenstein SR, Cohn JF, Shalaby TM, Kartan R 1993 Reliability and validity of an infant gastroesophageal reflux questionnaire. Clin Pediatr (Phila) 32:472-484.
  - Orenstein SR, Shalaby TM, Cohn JF 1996 Reflux symptoms in 100 normal infants: diagnostic validity of the infant gastroesophageal reflux questionnaire. Clin Pediatr (Phila) 35:607-614.
  - Di Lorenzo C, Orenstein S 2002 Fundoplication: friend or foe? J Pediatr Gastroenterol Nutr 34:117-124.
  - El-Serag HB, Gilger M, Kuebeler M, Rabeneck L 2001 Extraesophageal associations of gastroesophageal reflux disease in children without neurologic defects. Gastroenterology 121:1294-1299.
  - Orenstein SR, Di Lorenzo C 2001 Postfundoplication Complications in Children. Curr Treat Options Gastroenterol 4:441-449.
  - Orenstein SR, Hassall E 2007 Infants and proton pump inhibitors: tribulations, no trials. J Pediatr Gastroenterol Nutr 45:395-398.
  - Martin JA 2011 Preterm births - United States, 2007. MMWR Surveill Summ 60 Suppl:78-79.
  - Fass R, Naliboff B, Higa L, Johnson C, Kodner A, Munakata J, Ngo J, Mayer EA 1998 Differential effect of long-term esophageal acid exposure on mechanosensitivity and chemosensitivity in humans.Gastroenterology 115:1363-1373.
  - Broussard DL, Altschuler SM 2000 Central integration of swallow and airway-protective reflexes. Am J Med 108 Suppl 4a:62S-67S.
  - Hirano I, Gilliam J, Goyal RK 2000 Clinical and manometric features of the lower esophageal muscular ring. Am J Gastroenterol 95:43-49.
  - Sivarao DV, Goyal RK 2000 Functional anatomy and physiology of the upper esophageal sphincter. Am J Med 108 Suppl 4a:27S-37S.
  - Altschuler SM 2001 Laryngeal and respiratory protective reflexes. Am J Med 111 Suppl 8A:90S-94S.
  - Li BU, Altschuler SM, Berseth CL, Di Lorenzo C, Rudolph CD, Scott RB 2002 Research agenda for pediatric gastroenterology, hepatology and nutrition: motility disorders and functional gastrointestinal disorders. Report of the North American Society for Pediatric Gastroenterology, Hepatology and Nutrition for the Children's Digestive Health and Nutrition Foundation. J Pediatr Gastroenterol Nutr 35Suppl 3:S263-267.
  - Boyle JT, Co J, Davidson G, Freston J, Nelson S, Tolia V, Werlin SL 2003 Do children with gastroesophageal reflux become adults with gastroesophageal reflux? What is the role of Acid suppression in children? J Pediatr Gastroenterol Nutr 37 Suppl 1:S65-68.
  - Cao W, Sohn UD, Bitar KN, Behar J, Biancani P, Harnett KM 2003 MAPK mediates PKC-dependent contraction of cat esophageal and lower esophageal sphincter circular smooth muscle. Am J Physiol Gastrointest Liver Physiol 285:G86-95.
  - Sarkar S, Thompson DG, Woolf CJ, Hobson AR, Millane T, Aziz Q 2004 Patients with chest pain and occult gastroesophageal reflux demonstrate visceral pain hypersensitivity which may be partially responsive to acid suppression. Am J Gastroenterol 99:1998-2006.
  - Di Fiore JM, Arko M, Whitehouse M, Kimball A, Martin RJ 2005 Apnea is not prolonged by acid gastroesophageal reflux in preterm infants. Pediatrics 116:1059-1063.
  - Mittal RK, Liu J 2005 Flow across the gastro-esophageal junction: lessons from the sleeve sensor on the nature of anti-reflux barrier. Neurogastroenterol Motil 17:187-190.
  - Vandenplas Y 2005 Gastroesophageal reflux: medical treatment. J Pediatr Gastroenterol Nutr 41 Suppl1:S41-42.
  - Guillet R, Stoll BJ, Cotten CM, Gantz M, McDonald S, Poole WK, Phelps DL 2006 Association of H2- blocker therapy and higher incidence of necrotizing enterocolitis in very low birth weight infants. Pediatrics 117:e137-142.
  - Martin RJ, Hibbs AM 2006 Diagnosing gastroesophageal reflux in preterm infants. Pediatrics 118:793-794.
  - Zerbib F, Roman S, Ropert A, des Varannes SB, Pouderoux P, Chaput U, Mion F, Verin E, Galmiche JP, Sifrim D 2006 Esophageal pH-impedance monitoring and symptom analysis in GERD: a study in patients off and on therapy. Am J Gastroenterol 101:1956-1963.
  - Havemann BD, Henderson CA, El-Serag HB 2007 The association between gastro-oesophageal reflux disease and asthma: a systematic review. Gut 56:1654-1664.
  - Slocum C, Hibbs AM, Martin RJ, Orenstein SR 2007 Infant apnea and gastroesophageal reflux: a critical review and framework for further investigation. Curr Gastroenterol Rep 9:219-224.
  - Castell DO 2008 Reflux testing in the 21st century: is there a role for pH only? Clin Gastroenterol Hepatol 6:840-841.
  - Hassall E, Owen D 2008 Long-term use of PPIs in children: we have questions. Dig Dis Sci 53:1158-1160, author reply 1161-1152.
  - Kahrilas PJ 2008 Clinical practice. Gastroesophageal reflux disease. N Engl J Med 359:1700-1707.
  - Kahrilas PJ, Shaheen NJ, Vaezi MF 2008 American Gastroenterological Association Institute technical review on the management of gastroesophageal reflux disease. Gastroenterology 135:1392-1413,1413 e1391-1395.
  - Kahrilas PJ, Sifrim D 2008 High-resolution manometry and impedance-pH/manometry: valuable tools in clinical and investigational esophagology. Gastroenterology 135:756-769
  - Lang IM, Haworth ST, Medda BK, Roerig DL, Forster HV, Shaker R 2008 Airway responses to esophageal acidification. Am J Physiol Regul Integr Comp Physiol 294:R211-219.
  - Orenstein SR, McGowan JD 2008 Efficacy of conservative therapy as taught in the primary care setting for symptoms suggesting infant gastroesophageal reflux. J Pediatr 152:310-314.
  - Rommel N, Davidson G, Cain T, Hebbard G, Omari T 2008 Videomanometric evaluation of pharyngo- oesophageal dysmotility in children with velocardiofacial syndrome. J Pediatr Gastroenterol Nutr 46:87-91.
  - Tutuian R, Vela MF, Hill EG, Mainie I, Agrawal A, Castell DO 2008 Characteristics of symptomatic reflux episodes on Acid suppressive therapy. Am J Gastroenterol 103:1090-1096.
  - Di Fiore JM, Arko M, Churbock K, Hibbs AM, Martin RJ 2009 Technical limitations in detection of gastroesophageal reflux in neonates. J Pediatr Gastroenterol Nutr 49:177-182.
  - El-Serag HB, Fitzgerald S, Richardson P 2009 The extent and determinants of prescribing and adherence with acid-reducing medications: a national claims database study. Am J Gastroenterol 104:2161-2167.
  - Fass R 2009 Clinical implications of chronic therapy for GERD. Preface. Am J Gastroenterol 104 Suppl 2:S1.
  - Gasiorowska A, Navarro-Rodriguez T, Wendel C, Krupinski E, Perry ZH, Koenig K, Moty B, Powers J, Fass R 2009 Comparison of the degree of duodenogastroesophageal reflux and acid reflux between patients who failed to respond and those who were successfully treated with a proton pump inhibitor once daily. Am J Gastroenterol 104:2005-2013.
  - Kuribayashi S, Massey BT, Hafeezullah M, Perera L, Hussaini SQ, Tatro L, Darling RJ, Franco R, Shaker R 2009 Terminating motor events for TLESR are influenced by the presence and distribution of refluxate. Am J Physiol Gastrointest Liver Physiol 297:G71-75.
  - Loots CM, Benninga MA, Davidson GP, Omari TI 2009 Addition of pH-impedance monitoring to standard pH monitoring increases the yield of symptom association analysis in infants and children with gastroesophageal reflux. J Pediatr 154:248-252.
  - Omari TI 2009 Apnea-associated reduction in lower esophageal sphincter tone in premature infants. J Pediatr 154:374-378.
  - Orenstein SR, Hassall E, Furmaga-Jablonska W, Atkinson S, Raanan M 2009 Multicenter, double-blind, randomized, placebo-controlled trial assessing the efficacy and safety of proton pump inhibitor lansoprazole in infants with symptoms of gastroesophageal reflux disease. J Pediatr 154:514-520 e514.
  - Richter JE 2009 Con: Impedance-pH testing does not commonly alter management of GERD. Am J Gastroenterol 104:2667-2669.
  - Sherman PM, Hassall E, Fagundes-Neto U, Gold BD, Kato S, Koletzko S, Orenstein S, Rudolph C,Vakil N, Vandenplas Y 2009 A global, evidence-based consensus on the definition of gastroesophageal reflux disease in the pediatric population. Am J Gastroenterol 104:1278-1295; quiz 1296.
  - van Wijk MP, Benninga MA, Omari TI 2009 Role of the multichannel intraluminal impedance technique in infants and children. J Pediatr Gastroenterol Nutr 48:2-12.
  - Boeckxstaens GE, Beaumont H, Mertens V, Denison H, Ruth M, Adler J, Silberg G, Sifrim D 2010Effects of lesogaberan on reflux and lower esophageal sphincter function in patients with gastroesophageal reflux disease. Gastroenterology 139:409-417.
  - Jiang Y, Sandler B, Bhargava V, Mittal RK 2011 Anti-Reflux Action of Nissen Fundoplication and Stretch Sensitive Mechanism of Lower Esophageal Sphincter Relaxation. Gastroenterology. 140:442-449.
  - Lacy BE, Weiser K, Chertoff J, Fass R, Pandolfino JE, Richter JE, Rothstein RI, Spangler C, Vaezi MF 2010 The diagnosis of gastroesophageal reflux disease. Am J Med 123:583-592.
  - Orenstein SR 2010 Symptoms and Reflux in Infants: Infant Gastroesophageal Reflux Questionnaire Revised (I-GERQ-R)-Utility for Symptom Tracking and Diagnosis. Curr Gastroenterol Rep.112:431-436.
  - Rieder F, Biancani P, Harnett K, Yerian L, Falk GW 2010 Inflammatory mediators in gastroesophageal reflux disease: impact on esophageal motility, fibrosis, and carcinogenesis. Am J Physiol Gastrointest Liver Physiol 298:G571-581.
  - Salvatore S, Arrigo S, Luini C, Vandenplas Y 2010 Esophageal Impedance in Children: Symptom- Based Results. J Pediatr. 157:949-954 e941-942.
  - Thakkar K, Boatright RO, Gilger MA, El-Serag HB 2010 Gastroesophageal reflux and asthma in children: a systematic review. Pediatrics 125:e925-930.
  - van Wijk MP, Benninga MA, Davidson GP, Haslam R, Omari TI 2010 Small volumes of feed can trigger transient lower esophageal sphincter relaxation and gastroesophageal reflux in the right lateral position in infants. J Pediatr 156:744-748, 748 e741.
  - Alstermark C, Amin K, Dinn SR, Elebring T, Fjellstrom O, Fitzpatrick K, Geiss WB, Gottfries J, Guzzo PR, Harding JP, Holmen A, Kothare M, Lehmann A, Mattsson JP, Nilsson K, Sunden G, Swanson M, von Unge S, Woo AM, Wyle MJ, Zheng X 2008 Synthesis and pharmacological evaluation of novel gamma-aminobutyric acid type B (GABAB) receptor agonists as gastroesophageal reflux inhibitors. Med Chem 51:4315-4320.
  - Cucchiara S, Franco MT, Terrin G, Spadaro R, di Nardo G, Iula V 2000 Role of drug therapy in the treatment of gastro-oesophageal reflux disorder in children. Paediatr Drugs 2:263-272.
  - DiPalma JR 1990 Metoclopramide: a dopamine receptor antagonist. Am Fam Physician 41:919-924.
  - Fass R, Bautista J, Janarthanan S 2003 Treatment of gastroesophageal reflux disease. Clin Cornerstone 5:18-29; discussion 30-11.
  - Gold BD, Freston JW 2002 Gastroesophageal reflux in children: pathogenesis, prevalence, diagnosis, and role of proton pump inhibitors in treatment. Paediatr Drugs 4:673-685.
  - Hassall E, Kerr W, El-Serag HB 2007 Characteristics of children receiving proton pump inhibitors continuously for up to 11 years duration. J Pediatr 150:262-267, 267 e261.
  - Hegar B, Alatas S, Advani N, Firmansyah A, Vandenplas Y 2009 Domperidone versus cisapride in the treatment of infant regurgitation and increased acid gastro-oesophageal reflux: a pilot study. Acta Paediatr 98:750-755.
  - Kahrilas PJ, Quigley EM, Castell DO, Spechler SJ 2000 The effects of tegaserod (HTF 919) on oesophageal acid exposure in gastro-oesophageal reflux disease. Aliment Pharmacol Ther 14:1503-1509.
  - Lehmann A 2009 GABAB receptors as drug targets to treat gastroesophageal reflux disease. Pharmacol Ther 122:239-245.
  - Lehmann A, Jensen JM, Boeckxstaens GE 2010 GABAB receptor agonism as a novel therapeutic modality in the treatment of gastroesophageal reflux disease. Adv Pharmacol 58:287-313.
  - Omari T, Lundborg P, Sandstrom M, Bondarov P, Fjellman M, Haslam R, Davidson G 2009Pharmacodynamics and systemic exposure of esomeprazole in preterm infants and term neonates with gastroesophageal reflux disease. J Pediatr 155:222-228.
  - Omari TI, Benninga MA, Sansom L, Butler RN, Dent J, Davidson GP 2006 Effect of baclofen on esophagogastric motility and gastroesophageal reflux in children with gastroesophageal reflux disease: a randomized controlled trial. J Pediatr 149:468-474.
  - Pehlivanov N, Sarosiek I, Whitman R, Olyaee M, McCallum R 2002 Effect of cisapride on nocturnal transient lower oesophageal sphincter relaxations and nocturnal gastro-oesophageal reflux in patients with oesophagitis: a double-blind, placebo-controlled study. Aliment Pharmacol Ther 16:743-747.
  - Richter JE, Castell DO 1981 Current approaches in the medical treatment of oesophageal reflux. Drugs 21:283-291.
  - Robinson M, Decktor DL, Maton PN, Sabesin S, Roufail W, Kogut D, Roberts W, McCullough A, Pardol P, Saco L, et al. 1993 Omeprazole is superior to ranitidine plus metoclopramide in the short-term treatment of erosive oesophagitis. Aliment Pharmacol Ther 7:67-73.
  - Talley NJ 1992 Review article: 5-hydroxytryptamine agonists and antagonists in the modulation of gastrointestinal motility and sensation: clinical implications. Aliment Pharmacol Ther 6:273-289.
  - Tutuian R, Mainie I, Allan R, Hargreaves K, Agrawal A, Freeman J, Gale J, Castell DO 2006 Effects of a 5-HT(4) receptor agonist on oesophageal function and gastro-oesophageal reflux: studies using combined impedance-manometry and combined impedance-pH. Aliment Pharmacol Ther 24:155-162.
  - 2003 Apnea, sudden infant death syndrome, and home monitoring. Pediatrics 111:914-917.
  - Hunt CE, Lesko SM, Vezina RM, McCoy R, Corwin MJ, Mandell F, Willinger M, Hoffman HJ, Mitchell, AA 2003 Infant sleep position and associated health outcomes. Arch Pediatr Adolesc Med 157:469-474.
  - Omari TI, Rommel N, Staunton E, Lontis R, Goodchild L, Haslam RR, Dent J, Davidson GP 2004 Paradoxical impact of body positioning on gastroesophageal reflux and gastric emptying in the premature neonate. J Pediatr 145:194-200.
  - Corvaglia L, Rotatori R, Ferlini M, Aceti A, Ancora G, Faldella G 2007 The effect of body positioning on gastroesophageal reflux in premature infants: evaluation by combined impedance and pH monitoring. J Pediatr 151:591-596, 596 e591.
  - Martin RJ, Di Fiore JM, Hibbs AM 2007 Gastroesophageal reflux in preterm infants: is positioning the answer? J Pediatr 151:560-561.
  - Vandenplas Y, De Schepper J, Verheyden S, Devreker T, Franckx J, Peelman M, Denayer E, Hauser B 2010 A preliminary report on the efficacy of the Multicare AR-Bed in 3-week-3-month-old infants on regurgitation, associated symptoms and acid reflux. Arch Dis Child 95:26-30.
  - **Jadcherla SR**, Wang M, Gupta A, Fernandez SA, Erdman S, DiLorenzo C 2009 Pathophysiology based individualized approach to the feeding management of the complex ICU neonate: Is this the HolyGrail? Gastroenterol 136:A-503.
  - **Jadcherla SR**, Wang M, Gupta A, SA F, Erdman S, Dilorenzo C 2009 Innovative personalized pathophysiology based feeding management improves feeding outcomes and quality of life in the complex ICU neonate: A novel approach for the future? Neurogastroenterol Motil 24:A-77.
  - **Jadcherla SR**, Duong HQ, Hoffmann RG, Shaker R 2003 Esophageal body and upper esophageal sphincter motor responses to esophageal provocation during maturation in preterm newborns. J Pediatr 143:31-38.
  - van Wijk MP, Benninga MA, Dent J, Lontis R, Goodchild L, McCall LM, Haslam R, Davidson GP, Omari, T 2007 Effect of body position changes on postprandial gastroesophageal reflux and gastric emptying in the healthy premature neonate. J Pediatr 151:585-590, 590 e581-582.
  - Gunasekaran TS, Hassall EG 1993 Efficacy and safety of omeprazole for severe gastroesophageal reflux in children. J Pediatr 123:148-154.
  - Quercia RA, Fan C, Liu X, Chow MS 1997 Stability of omeprazole in an extemporaneously prepared oral liquid. Am J Health Syst Pharm 54:1833-1836.
  - Israel DM, Hassall E 1998 Omerprazole and other proton pump inhibitors: pharmacology, efficacy, and safety, with special reference to use in children. J Pediatr Gastroenterol Nutr 27:568-579.
  - DiGiacinto JL, Olsen KM, Bergman KL, Hoie EB 2000 Stability of suspension formulations of lansoprazole and omeprazole stored in amber-colored plastic oral syringes. Ann Pharmacother 34:600-605.
  - Hassall E, Israel D, Shepherd R, Radke M, Dalvag A, Skold B, Junghard O, Lundborg P 2000 Omeprazole for treatment of chronic erosive esophagitis in children: a multicenter study of efficacy, safety, tolerability and dose requirements. International Pediatric Omeprazole Study Group. J Pediatr 137:800-807.
  - Moore DJ, Tao BS, Lines DR, Hirte C, Heddle ML, Davidson GP 2003 Double-blind placebo-controlled trial of omeprazole in irritable infants with gastroesophageal reflux. J Pediatr 143:219-223.
  - Barron JJ, Tan H, Spalding J, Bakst AW, Singer J 2007 Proton pump inhibitor utilization patterns in infants. J Pediatr Gastroenterol Nutr 45:421-427.
  - Omari TI, Haslam RR, Lundborg P, Davidson GP 2007 Effect of omeprazole on acid gastroesophageal reflux and gastric acidity in preterm infants with pathological acid reflux. J Pediatr Gastroenterol Nutr44:41-44.
  - Anderson DM 2002 Feeding the ill or preterm infant. Neonatal Netw 21:7-14.
  - Caple J, Armentrout D, Huseby V, Halbardier B, Garcia J, Sparks JW, Moya FR 2004 Randomized, controlled trial of slow versus rapid feeding volume advancement in preterm infants. Pediatrics114:1597-1600.
  - Kashyap S 2007 Enteral intake for very low birth weight infants: what should the composition be? Semin Perinatol 31:74-82.
  - Kuschel CA, Evans N, Askie L, Bredemeyer S, Nash J, Polverino J 2000 A randomized trial of enteral feeding volumes in infants born before 30 weeks' gestation. J Paediatr Child Health 36:581-586.
  - Romera G, Figueras J, Rodriguez-Miguelez JM, Ortega J, Jimenez R 2004 Energy intake, metabolic balance and growth in preterm infants fed formulas with different nonprotein energy supplements. J Pediatr Gastroenterol Nutr 38:407-413.
  - Valentine CJ, Griffin IJ, Abrams SA 2003 Nutritional Support in Children. CRC Press.1996 American Gastroenterological Association medical position statement: guidelines on the use of esophageal pH recording. Gastroenterology 110:1981.
  - Engle WA 2004 Age terminology during the perinatal period. Pediatrics 114:1362-1364.
  - Orenstein SR 2010 Symptoms and reflux in infants: Infant Gastroesophageal Reflux Questionnaire Revised (I-GERQ-R)--utility for symptom tracking and diagnosis. Curr Gastroenterol Rep 12:431-436.
  - Vernacchio L, Corwin MJ, Lesko SM, Vezina RM, Hunt CE, Hoffman HJ, Willinger M, Mitchell AA 2003 Sleep position of low birth weight infants. Pediatrics 111:633-640.
  - Wenzl TG, Silny J, Schenke S, Peschgens T, Heimann G, Skopnik H 1999 Gastroesophageal reflux and respiratory phenomena in infants: status of the intraluminal impedance technique. J Pediatr Gastroenterol Nutr 28:423-428.
  - Lopez-Alonso M, Moya MJ, Cabo JA, Ribas J, del Carmen Macias M, Silny J, Sifrim D 2006 Twenty- four-hour esophageal impedance-pH monitoring in healthy preterm neonates: rate and characteristics of acid, weakly acidic, and weakly alkaline gastroesophageal reflux. Pediatrics 118:e299-308.
  - Francavilla R, Magista AM, Bucci N, Villirillo A, Boscarelli G, Mappa L, Leone G, Fico S, Castellaneta S, Indrio F, Lionetti E, Moramarco F, Cavallo L 2010 Comparison of esophageal pH and multichannel intraluminal impedance testing in pediatric patients with suspected gastroesophageal reflux. J Pediatr Gastroenterol Nutr 50:154-160.
  - Rosen R, Levine P, Lewis J, Mitchell P, Nurko S 2010 Reflux events detected by pH-MII do not determine fundoplication outcome. J Pediatr Gastroenterol Nutr 50:251-255.
  - Pilic D, Frohlich T, Noh F, Pappas A, Schmidt-Choudhury A, Kohler H, Skopnik H, Wenzl TG 2011 Detection of gastroesophageal reflux in children using combined multichannel intraluminal impedance and pH measurement: data from the German Pediatric Impedance Group. J Pediatr 158:650-654 e651.
  - Corvaglia L, Zama D, Spizzichino M, Aceti A, Mariani E, Capretti MG, Galletti S, Faldella G 2011 The frequency of apneas in very preterm infants is increased after non-acid gastro-esophageal reflux. Neurogastroenterol Motil 23:303-e152.
  - Ghezzi M, Silvestri M, Guida E, Pistorio A, Sacco O, Mattioli G, Jasonni V, Rossi GA 2011 Acid and weakly acid gastroesophageal refluxes and type of respiratory symptoms in children. Respir Med 105:972-978.
  - Luthold SC, Rochat MK, Bahler P 2010 Disagreement between symptom-reflux association analysis parameters in pediatric gastroesophageal reflux disease investigation. World J Gastroenterol 16:2401-2406.
  - Omari TI, Schwarzer A, vanWijk MP, Benninga MA, McCall L, Kritas S, Koletzko S, Davidson GP 2011Optimisation of the reflux-symptom association statistics for use in infants being investigated by 24- hour pH impedance. J Pediatr Gastroenterol Nutr 52:408-413.
  - Rosen R, Nurko S 2004 The importance of multichannel intraluminal impedance in the evaluation of children with persistent respiratory symptoms. Am J Gastroenterol 99:2452-2458.
  - Slocum C, Arko M, Di Fiore J, Martin RJ, Hibbs AM 2009 Apnea, bradycardia and desaturation in preterm infants before and after feeding. J Perinatol 29:209-212.
  - Wiener GJ, Richter JE, Copper JB, Wu WC, Castell DO 1988 The symptom index: a clinically important parameter of ambulatory 24-hour esophageal pH monitoring. Am J Gastroenterol 83:358-361.
  - Lam HG, Breumelhof R, Roelofs JM, Van Berge Henegouwen GP, Smout AJ 1994 What is the optimal time window in symptom analysis of 24-hour esophageal pressure and pH data? Dig Dis Sci 39:402-409.
  - Weusten BL, Roelofs JM, Akkermans LM, Van Berge-Henegouwen GP, Smout AJ 1994 The symptom- association probability: an improved method for symptom analysis of 24-hour esophageal pH data. Gastroenterology 107:1741-1745.
  - **Jadcherla SR**, Shaker R 2001 Esophageal and upper esophageal sphincter motor function in babies. Am J Med 111 Suppl 8A:64S-68S.
  - **Jadcherla SR** 2002 Gastroesophageal reflux in the neonate. Clin Perinatol 29:135-158.
  - **Jadcherla SR** 2003 Manometric evaluation of esophageal-protective reflexes in infants and children. Am J Med 115 Suppl 3A:157S-160S.
  - Holm S 1979 A simple sequentially rejective multiple test procedure Scan J Statist 6:65-70
  - Lan KKG, Demets DL 1983 Discrete Sequential Boundaries for Clinical Trials. Biometrika 70:659-663.

# Study Endpoints

- Primary Outcome
  - **Clinical outcome of feeding success for those infants transitioning to oral feeds at inception**.
    - The primary endpoint is the feeding success defined as achieving full oral feeds (defined as no need for tube feeds to maintain hydration and nutrition) and/or a >/= 6-point decrease from baseline I-GERQ-R. The time frame is up to 5 weeks after enrollment.
  - **Clinical outcome of feeding success for those infants on full oral feeds at inception*.***
    - The primary endpoint is the feeding success defined as maintaining full oral feeds and a >/= 6-point decrease from baseline I-GERQ-R. The time frame is up to 5 weeks after enrollment.
- Secondary Outcomes
  - Clinical Outcomes
    - Length of hospital stay
    - Growth outcomes during the study period and up to 1 year
    - Independent feeding skills over time up to 1 year
    - Respiratory support during the study period and at discharge
    - Developmental outcomes with Bayley’s scores at 2 years
    - Individual differences in I-GERQ-R questionnaire
  - Motility Outcomes
    - Mechanisms of aerodigestive clearance and esophageal provocation are evaluated based on esophageal motility studies at inception and after 5 weeks.
  - pH Impedance outcomes
    - 24-hour pH impedance results at inception of study and then 5 weeks later after treatment completed

# Study Intervention/Investigational Agent

- - Those subjects randomized into the study group will follow innovative feeding strategy for 5 weeks. The innovative feeding strategy is as follows:
- Feeding Volume <140 ml/kg/d
- Feeding duration over 30 minutes
- Right lateral feeding position
- Supine position postprandial

# Procedures Involved*

- 1. **Study design:**
- Study design and setting: This is a Single Center Prospective Randomized, Blinded Controlled Trial. Consented subjects will undergo 1:1 randomization into either innovative feeding strategy and standard therapy arms.
- Patient Population: Potential study subjects are those that are referred by their attending physician for evaluation and management of potential GERD, the key symptoms being irritability and arching, gagging, choking, coughing, aspiration, life threatening events, swallowing problems, and/or failure to thrive. I-GERQ-R questionnaire will be completed as per guidelines. Each patient must have a signed consent from parent/legal guardian prior to initiation of screening for the trial.
- Inclusion Criteria: Hospitalized convalescing infants with aerodigestive symptoms or admitted for GERD symptoms, < 42 weeks GA. Infants will be included if PMA at time of study is ≥ 34 wks age and < 60 wks. Subjects must be on full enteral feeds defined as ≥ 150mL/k/day. Infants must be breathing unaided, and nasal cannula oxygen up to 1 LPM is allowed. In subjects receiving empiric prokinetics or acid suppressive agents, the medications need to be stopped for ≥ 72 hr prior to evaluation.
- Exclusion Criteria: Infants with known genetic, metabolic or syndromic disease, neurological diseases such as Grade III or IV intra ventricular hemorrhage or perinatal asphyxia, GI malformations and surgical GI conditions.
- Tests and procedures
  - Twenty-four-hour pH and impedance ordered diagnostically for suspected GERD
  - If RI >3 and infant meets all above criteria, infant randomized into conventional or study group
  - Esophageal manometry performed
  - 4 weeks of the study intervention for the study group and standard treatment for the conventional group while collecting data on symptoms, feeding position, duration and volume
  - Omeprazole stopped after 4-week course.
  - Repeat esophageal manometry and pH impedance testing done after 1 week off omeprazole (at week 5). If the providers/parents are resistant or refuse to stop the medication, repeat testing will still be done, and the alteration will be noted.
- Overall Study Endpoints: Primary clinical outcome occurs at time 2 study or at discharge if time 2 study not completed. Secondary study outcomes occur at time 2 studies. Secondary clinical outcome measures (growth characteristics, development and long term feeding outcomes) will be collected up to 24 months.
- Methods to overcome potential confounders: Data will be collected from EPIC documentation, feeding diaries, parent interviews and follow up appointments. While inpatient, these are documented regularly. After discharge, we will be dependent on parents to provide the data.
- Removal and management of subject(s) from protocol: The investigator and/or the parent(s) have the right to withdraw from study at any time and opt for the standard of care. Parent(s) that withdraw their infants from the study therapy will be encouraged to continue to remain in the study for all follow up evaluations. Those who choose completely withdrawal from the study will be encouraged to follow the standard of care; wherein, response failures are followed for secondary outcomes until 18 months.
- Secondary Outcome Measures: (up to 24 months, for Bayleys developmental score) Secondary outcome measures (growth characteristics, development and long term feeding outcomes) will be collected up to 12 months.
  1. **Describe:**
     - 24-hour Esophageal pH/impedance testing and calculation of acid reflux index (ARI) is a standard clinical test. Subjects will be monitored 1:1 by trained staff not associated with the study for the duration of the test. This allows us to quantify the components of refluxate and associated symptom indices while assuring subject safety.
     - Manometry methods have been used concurrent with video, submental EMG, RIP, ECG, pulse-oximetry and nasal air flow to test basal and adaptive pharyngo-esophageal reflexes and sensory-motor characteristics of motility and to monitor safety Concurrent synchronized video recordings will be performed to further validate symptoms based on objective definition of the esophageal reflexes. Documentation of symptom markers can be validated by integrating manometry with respiratory inductance plethysmography and video.
     - Manometry and pH/impedance testing are both performed on a routine basis for clinical diagnostic purposes. The catheters needed to perform these studies are placed under strict guidelines by, in the instance of pH/impedance testing, a trained Registered Nurse or, in the case of manometry testing, a trained physician. The pH/impedance catheter placement is confirmed by x-ray.
- For the duration of both of these procedures, the subject is monitored by a trained RN, and vital signs (heart rate, breathing and oxygen saturation) are monitored by both visualization of subject and electronic monitoring.
- Equipment used to perform these procedures are commonly used in clinical diagnostic testing and are being used for FDA approved purposes.
- Omeprazole is prescribed to both groups and is the standard of care in the NICU.
- The source records that will be used to collect data about subjects will be EPIC records, patient diary logs for symptoms, I-GERQ-R questionnaires, feeding process, medication compliance, physician visits and clinical data.
  1. **Data to be collected**
- Patient diary logs for symptoms, feeding process, medication compliance, physician visits and clinical data.
- 24-hr pH data will be collected to characterize the frequency, acid reflux index, Vandenplas score, longest reflux event duration, and number longer than 5 min
- Basal manometry data will be collected to assess swallow frequency, resting UES and LES pressure, swallow propagation types, peristaltic velocity, frequency of symptoms and the causative mechanisms.
- Multimodal sensory motor testing involves mid-esophageal provocation with graded infusions (0.1 to 5 ml) of air (to stimulate mechanoreceptors), apple juice (pH 3.7, to stimulate acid-sensitive receptors), and sterile water (pH 7.0, as control stimulus), and examine the changes in sensory-motor characteristics of aerodigestive reflexes pertinent to secondary peristalsis or deglutition response, esophago-UES contractile reflex, LES-relaxation reflex, and symptoms during the stimulation. This protocol will be performed in both experimental and standard therapy groups at time-1 and time-2 (where feasible).
- Basal and adaptive pharyngeal reflexes with air and water infusions are measured at time-1 and time-2 (where feasible).
- Subjects will be monitored up to one year through electronic medical records (EPIC) for number of hospital readmissions, number of clinic visits, growth metrics and medication use.

**6.4 Long term follow up**

- Long term follow-up will include feeding method, growth characteristics and development up to 18 months of age.

# Sharing of Results with Subjects*

Results of pH/impedance testing will be shared with care team and/or parents at the time of testing. Results of manometry testing will be conveyed verbally to parents and/or the care team at the time of testing along with any incidental findings that may impact subject’s care.

# Study Timelines*

The study procedures (pH/impedance and manometry) will be performed two times with 5 weeks between.

Omeprazole will be given for 4 weeks, with a 1-week washout period prior to the performance of the second studies.

# Inclusion and Exclusion Criteria*

Inclusion criteria:

- Hospitalized infants with aero-digestive symptoms or were admitted for GERD symptoms
- ≥ 34 weeks PMA and ≤ 60 weeks PMA (PMA= GA+ Chronological age)
- Enterally fed infants (PO or NG)
- Average daily fluid of 150 to 170 ml/kg/day at time of study
- Supplemental oxygen of ≤ 1 LPM by nasal cannula
- Subjects receiving empiric prokinetics or acid suppressive agents, the medications will be stopped for ≥ 72 hr prior to evaluation

Exclusion criteria:

- Known genetic, metabolic or syndromic disease
- Grade III or IV IVH or intra-cranial hemorrhage, perinatal asphyxia
- GI malformations or surgical GI conditions

Infants who are hospitalized in the NICU at NCH main campus and have undergone pH/impedance testing will be screened for the above inclusion and exclusion criteria. Our population will include individuals who are not yet adults (infants)

# Vulnerable Populations:

**10.1** This research complies with 45 CFR 46, as it includes only human infants up to 6 months old. As this study involves slightly greater than minimal risk, as regulated in 45 CFR 46 Subpart D, all subjects for this study will have consent of at least one parent.

- The Neonatal and Infant Feeding Disorders (NIFD) Program is directed by the PI, Dr. Sudarshan Jadcherla who is a Neonatologist with Pediatric GI experience and is a recognized GI motility expert. The NIFD program is a nationally recognized clinical program supported by the NIH and is well equipped to conduct clinical studies such as proposed in this application. In addition, there is a team of dedicated trained nurse coordinators who are competent and comfortable with performing these procedures on preterm infants as well as a neonatal nurse practitioner. The team also includes trained technical personnel affiliated to this program who perform these studies on a regular basis for diagnostic purposes in this same population.

# Local Number of Subjects

- N=120 subjects
- This is a single center trial, all subjects will be recruited at Nationwide Children’s Hospital

# Recruitment Methods

- 1. Infant’s referred to the Neonatal and Infant Feeding Disorders Program at Nationwide Children’s Hospital who have a pH/impedance study will be screened.
  2. RN Coordinators will identify and screen potential subjects
  3. Parents of infants will be offered $20.00 for each study visit completed. Payment is made in the form a debit card with payment loaded to it following the visit.

#

# Withdrawal of Subjects*

- If a subject develops any condition outlined in the exclusion criteria (i.e. surgical or neurological conditions) they will be withdrawn
- Subjects will be withdrawn if there are any safety concerns
- Parents can choose to withdraw their infant at any time during the study period, but some data collection will continue.

#

# Risks to Subjects*

This study includes the risk associated with the nasal placement of the manometry probe and pH/impedance catheter. These risks are similar to the risks associated with the placement of a nasogastric feeding tube in infants which is frequently done for tube feeding infants both in the hospital and sometimes at home

# Potential Benefits to Subjects*

Infants can benefit from evaluation careful observation of GERD feeding and breathing problems. The esophageal manometry may provide added information about the infant’s feeding problem and those results will be reported to the parents and providers.

#

# Data Management* and Confidentiality

- 1. **Data analysis plan: See above statistical analysis**
  2. **Data safety plan**:
- All data will be stored on password protected computers accessible by trained Research assistants within the lab. Redcap will be used to store data. All paper CRFs are stored in locked cabinets with access limited to lab staff.
- Data will be verified by at least two specially trained study team members.

# Provisions to Monitor the Data to Ensure the Safety of Subjects*

- - Subjects are monitored throughout study procedures by a trained Registered nurse
  - Meetings of the Data Safety Monitoring Board will take place on a quarterly basis.
  - The study is overseen by the IRB at NCH and annual reports will be submitted
  - A Data safety monitoring plan will be implemented with reports given to the DSMB and the IRB and NIDDK when appropriate.
  - Subjects who remain hospitalized throughout the study period will be monitored by lab staff via review of their electronic medical record and review of bedside documentation
  - Subjects who have been discharged to home will be monitored by weekly telephone communication with parents and caregivers
  - Parents will be told whom to contact in case of concerns

#

# Provisions to Protect the Privacy Interests of Subjects

- - Parents will have the choice of whether to participate and share information about their child.
  - The research team will access electronic records for follow up data.

# Compensation for Research-Related Injury

- - Research related injury will not be compensated
  - Language from consent:
    - If your child is hurt by the procedures that are part of the Study, you should seek medical treatment for the injuries and tell the Study Doctor as soon as possible at the number on the first page of this form. If it is an emergency, call 911 or go to the nearest emergency department. In most cases, this care will be billed to your health insurance company or whoever usually pays for your health care at the usual charges, but some insurance companies will not pay for care related to a study. If the care is provided at Nationwide Children's Hospital, we make no commitment to pay for the medical care provided to you. No funds have been set aside to compensate you in the event of injury. If no one else pays for your care, you may have to pay for the cost of this care. This does not mean that you give up any of your legal rights to seek compensation for your injuries

# Economic Burden to Subjects

- - None

# Consent Process

- - Consent
    - Consent will take place in person with one parent (risk level 2) prior to the study taking place.
    - We will be following “SOP: Informed Consent Process for Research (HRP-090).”

**Non-English Speaking Subjects – if known, skip if not known**

- - - If subjects do not speak English are enrolled, the consent form will be translated in person by an interpreter speaking the language of the parent.

**Subjects who are not yet adults (infants, children, teenagers)**

- - - All subjects enrolled in this study will be infants.
    - Parental permission will can be obtained from one parent only as this study is level 2 risk.
    - Consent will not be obtained from individuals other than parents (i.e. foster parents, custodial agencies)
    - Consent will be obtained in person

# Process to Document Consent in Writing

- 1. We will be following “SOP: Written Documentation of Consent (HRP-091).

#

# Setting

- This research will be conducted at Nationwide Children’s Hospital, Columbus, Ohio
- Research procedures will be performed at Nationwide Children’s Hospital
- Safety monitoring will be conducted through the NCH IRB

#

# Multi-Site Research*

24.1 This is a single site study with collaboration from The Ohio State University Department of Biostatistics for assistance with data analysis. Dr. Wei is a Research Assistant Professor at the Department of Biostatistics at The Ohio State University College of Medicine and Public Health. She has expertise in clinical statistical modeling techniques. She has worked with the PI with sample size and statistical design for this study. She will provide consultation with analysis and design support. Dr. Wei works with the PI closely with regards to statistical outputs and manuscript writing.

24.2 Data is stored in REDCap where all of the study staff including Dr. Wei can access the data. Subject identifiers include Names, Birth Date, Discharge Date and Medical Record Numbers as identified in the consent form.

# ****24.0 Protected Health Information Recording****

1. **Indicate which subject identifiers will be recorded for this research.**

Name

Complete Address

Telephone or Fax Number

Social Security Number (do not check if only used for ClinCard)

Dates (treatment dates, birth date, date of death)

Email address, IP address or URL

Medical Record Number or other account number

Health Plan Beneficiary Identification Number

Full face photographic images and/or any comparable images (x-rays)

Account Numbers

Certificate/License Numbers

Vehicle Identifiers and Serial Numbers (e.g. VINs, License Plate Numbers)

Device Identifiers and Serial Numbers

Biometric identifiers, including finger and voice prints

Other number, characteristic or code that could be used to identify an individual

None (Complete De-identification Certification Form)

**2.0  Check the appropriate category and attach the required form* on the Local Site Documents, #3. Other Documents, page of the application.  (Choose one.)**

Patient Authorization will be obtained. (Include the appropriate HIPAA language (see Section 14 of consent template) in the consent form OR attach the HRP-900, HIPAA AUTHORIZATION form.)

Protocol meets the criteria for waiver of authorization. (Attach the HRP-901, WAIVER OF HIPAA AUTHORIZATION REQUEST form.)

Protocol is using de-identified information. (Attach the HRP-902, DE-IDENTIFICATION CERTIFICATION form.) (Checked "None" in 1.0 above)

Protocol involves research on decedents. (Attach the HRP-903, RESEARCH ON DECEDENTS REQUEST form.)

Protocol is using a limited data set and data use agreement. (Contact the Office of Technology Commercialization to initiate a Limited Data Use Agreement.

***Find the HIPAA forms in the IRB Website Library, Templates.**

**Attach the appropriate HIPAA form on the “Local Site Documents, #3. Other Documents”, page of the application.**

1. **How long will identifying information on each participant be maintained?**

**3.1 Throughout the study period and 18 months of follow up follow up, plus 10 years.**

1. **Describe any plans to code identifiable information collected about each participant.**

Each subject will be given a code known only to study staff and identifiable information will be kept separate from study data. Both will be stored in a secure area in a locked cabinet or in a computer database accessible only by study staff.

**Check each box that describes steps that will be taken to safeguard the confidentiality of information collected for this research:**

**X Research records will be stored in a locked cabinet in a secure location**

**X Research records will be stored in a password-protected computer file**

**X The list linking the assigned code number to the individual subject will be maintained separately from the other research data**

**X Only certified research personnel will be given access to identifiable subject information**

**5.0 Describe the provisions included in the protocol to protect the privacy interests of subjects, where "privacy interests" refer to the interest of individuals in being left alone, limiting access to them, and limiting access to their information. (This is not the same provision to maintain the confidentiality of data.**

**25.0 Confidential Health Information**

1. **Please mark all categories that reflect the nature of health information to be accessed and used as part of this research.**

Demographics (age, gender, educational level)

Diagnosis

Laboratory reports

Radiology reports

Discharge summaries

Procedures/Treatments received

Dates related to course of treatment (admission, surgery, discharge)

Billing information

Names of drugs and/or devices used as part of treatment

Location of treatment

Name of treatment provider

Surgical reports

Other information related to course of treatment

None

1. Please discuss why it is necessary to access and review the health information noted in your response above.

2.1 We will be looking at outcome variables for the first 18 months of life.

3.0 Is the health information to be accessed and reviewed the minimal necessary to achieve the goals of this research?  Yes  No

4.0 Will it be necessary to record information of a sensitive nature?  Yes  No

5.0 Do you plan to obtain a federally-issued Certificate of Confidentiality as a means of protecting the confidentiality of the information collected?  Yes  No
